# Alterations of mTOR signaling impact metabolic stress resistance in colorectal carcinomas with BRAF and KRAS mutations

**DOI:** 10.1038/s41598-018-27394-1

**Published:** 2018-06-15

**Authors:** Raphaela Fritsche-Guenther, Christin Zasada, Guido Mastrobuoni, Nadine Royla, Roman Rainer, Florian Roßner, Matthias Pietzke, Edda Klipp, Christine Sers, Stefan Kempa

**Affiliations:** 1 Max-Delbrueck-Center for Molecular Medicine in the Helmholtz Association (MDC), Berlin Institute of Health (BIH), Robert-Roessle-Str. 10, 13125 Berlin, Germany; 2 Max-Delbrueck-Center for Molecular Medicine in the Helmholtz Association (MDC), Berlin Institute for Medical Systems Biology (BIMSB), Robert-Roessle-Str. 10, 13125 Berlin, Germany; 3 Humboldt University Berlin, Theoretical Biophysics, Invalidenstraße 42, 10115 Berlin, Germany; 4 Charité Universitätsmedizin, Institute of Pathology, Chariteplatz 1, 10117 Berlin, Germany; 5 Beatson Institute, Switchback Road, Bearsden, Glasgow, G61 1BD United Kingdom

## Abstract

Metabolic reprogramming is as a hallmark of cancer, and several studies have reported that BRAF and KRAS tumors may be accompanied by a deregulation of cellular metabolism. We investigated how BRAF^V600E^ and KRAS^G12V^ affect cell metabolism, stress resistance and signaling in colorectal carcinoma cells driven by these mutations. KRAS^G12V^ expressing cells are characterized by the induction of glycolysis, accumulation of lactic acid and sensitivity to glycolytic inhibition. Notably mathematical modelling confirmed the critical role of MCT1 designating the survival of KRAS^G12V^ cells. Carcinoma cells harboring BRAF^V600E^ remain resistant towards alterations of glucose supply or application of signaling or metabolic inhibitors. Altogether these data demonstrate that an oncogene-specific decoupling of mTOR from AMPK or AKT signaling accounts for alterations of resistance mechanisms and metabolic phenotypes. Indeed the inhibition of mTOR in BRAF^V600E^ cells counteracts the metabolic predisposition and demonstrates mTOR as a potential target in BRAF^V600E^-driven colorectal carcinomas.

## Introduction

Colorectal tumors marked by the BRAF and KRAS oncogenes share some attributes that are also common in other tumor entities, including the epithelial to mesenchymal transition (EMT), differentiation, angiogenesis and adaptations of cellular metabolism^1^. But the mutations are mutually exclusive, suggesting that they promote transformation and cancer progression in the intestinal epithelium in distinct ways^1^.

Colorectal cancer cells frequently become addicted to oncogenic signals such as KRAS, which has led researchers to try to develop therapies that target them. So far such attempts based on KRAS have not been successful, but no specific inhibitor has been found^2^. In its absence, the effects of MEK inhibitors have been studied in tumors expressing mutated BRAF and KRAS; however, they led to tumor resistance through feedback and crosstalk mechanisms within the EGFR/MAPK and EGFR/PI3K signaling pathway^3–6^.

Metabolic deregulation is regarded as a hallmark of cancer^7^, and numerous studies have reported that BRAF or KRAS tumors may be accompanied by a reprogramming of cellular metabolism^8^. The oncogene-dependent upregulation of glycolysis leads to an increase in glucose consumption, the induction of *de novo* lipid synthesis and, as described years ago by Otto Warburg, the increased formation of lactic acid^8–12^. The high metabolic activity of cancer cells produces a gradient in the availability of nutrients, particularly glucose, and cellular signaling and the metabolic network needs to cooperate to adjust to the change.

Since the mechanisms by which metabolic alterations interact with signaling downstream from mutated BRAF and KRAS have not been completely elucidated, the aim of our study was to investigate the impact of BRAF^V600E^ and KRAS^G12V^ on tumor cell metabolism and signaling. We took an integrative approach that combined ELISA-based phosphoproteomics and mass spectrometry (MS)-based proteomics and pulse stable isotope resolved metabolomics (pSIRM)-derived data to analyze oncogene-dependent variations of the central carbon metabolism (CCM). We used the BRAF and KRAS wildtype CaCO2 colorectal carcinoma cell line, harboring Doxycycline inducible constructs expressing BRAF^V600E^ and KRAS^G12V^ as well as cell lines with naturally occurring BRAF^V600E^ (HT29) and KRAS^G12V^ (SW480) mutations. It is commonly accepted that the amount of glucose that is available differs between the layers of solid tumors. To replicate such areas we applied varying concentrations of glucose. We found that cells expressing BRAF^V600E^ and KRAS^G12V^ had similar morphologies and mitogenic signaling properties; however, their resistance mechanisms diverge and cause substantial differences in signaling to mTOR and glucose sensitivity.

Currently, KRAS and BRAF mutations are not seen as “only” altering signaling during the development of colorectal cancer. Tumors vary in their responses to treatments by inhibitors, developing resistance through mechanisms that provide different selective advantages. This means that attempts to find novel predictive markers and therapeutic options should not focus exclusively on the inhibition of signals, but needs to take the larger cellular context into account. Studying the combination of changes in signaling and metabolic networks that occur in cells as a result of the KRAS and BRAF oncogenes should provide insights into both fundamental tumor processes and the mechanisms by which they circumvent therapies.

## Results

### BRAF^V600E^ and KRAS^G12V^ induce similar physiological phenotypes, but different metabolic dependencies

The CaCO2 colorectal carcinoma cell line is an established *in vitro* model for the human intestinal epithelium. Cells harbor structural and functional characteristics that are similar to those of enterocytes and spontaneously differentiate under *in vitro* culture conditions^13^. The cell lines were treated with Doxycycline for a minimum of 7 days to provoke the sustainable expression of BRAF^V600E^ or KRAS^G12V^. A cell line containing an empty expression vector (CaCO2-control) was included as control and treated in parallel in all experiments. To exclude changes directly induced by Doxycycline two cell lines with naturally occurring BRAF^V600E^ (HT29) and KRAS^G12V^ (SW480) mutations were included.

**Figure 1:**
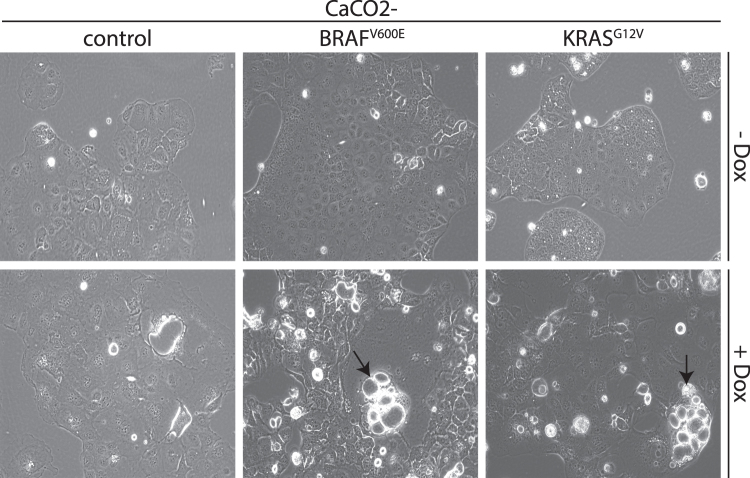
Expression of BRAF^V600E^ and KRAS^G12V^ resulted in cytoskeletal rearrangement and Mucin production. CaCO2-control, CaCO2-BRAF^V600E^ and CaCO2-KRAS^G12V^ cells cultivated with 1.0 g/L glucose were supplemented with Doxycycline for 16 days. Vacuoles containing Mucin are depicted by arrows. Pictures were taken with Zeiss Axio Z1 microscope using 200x magnification.

Cancer cells may adapt to changes in glucose concentrations by altering their morphology^14^. When we replicated this situation by changing the availability of glucose, this did not lead to glucose-induced morphological alterations in CaCO2-control, CaCO2-BRAF^V600E^ or CaCO2-KRAS^G12V^ cells (Fig. 1, Supplementary Figure 1A). High-throughput LC-MS (liquid chromatography coupled mass spectrometry) shotgun proteomics analysis allowed us to quantify the oncogene-dependent expression of EMT-related proteins. Comparisons revealed that CaCO2 cells expressing BRAF^V600E^ and KRAS^G12V^ regulate proteins e.g. Desmoplakin (DSP) involved in actin cytoskeleton remodeling, migration and adhesion in similar ways (Supplementary Figure 1B and 3A).

In addition to a loss of cell-cell contacts in these cells, we observed an oncogene-induced glucose-independent goblet-cell specific formation of vacuoles containing Mucin on the apical site of the vesicles (Fig. 1 and Supplementary Figure 1A). Mucin is known to play a major role in the pathogenesis of cancer^15^. We performed PAS (periodic acid-Schiff reaction) and alcian blue (AB) staining to specify the content of the vacuoles and detected a high amount of neutral and acid Mucin (Supplementary Figure 1C). Raised levels of MUC5AC, known to be specifically *de novo* expressed in colorectal carcinoma^16^ were observed in CaCO2-BRAF^V600E^ and CaCO2-KRAS^G12V^ cells (Supplementary Figure 1D).

**Figure 2:**
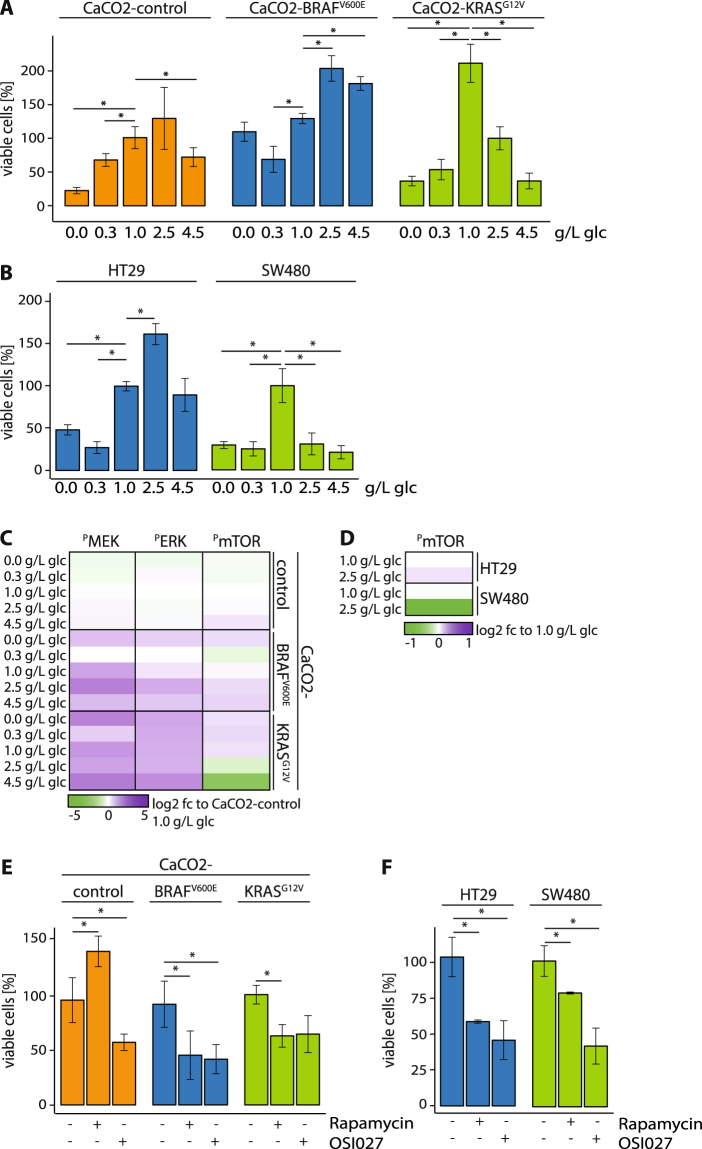
BRAF^V600E^ and KRAS^G12V^ induced glucose-dependent proliferation and alterations of signaling to mTOR. (**A**,**B**) CaCO2-control, CaCO2-BRAF^V600E^, CaCO2-KRAS^G12V^, HT29 and SW480 cells were cultivated with indicated glucose concentrations. Shown are viable cells (A: normalized to CaCO2-control cells 1.0 g/L glucose and B: normalized to 1.0 g/L glucose per cell line). Shown are standard deviation of (in minimum) n = 3 replicates. p < 0.05 was indicated with asterisk (unpaired two-tailed *t* Test). (**C**,**D)** Phosphorylation of signaling molecules were analyzed with ELISA bead-based phosphoproteomics technology (Luminex). Shown are log2 fold changes (fc, A: normalized to CaCO2-control cells 1.0 g/L glucose and B: normalized to 1.0 g/L glucose per cell line) of (in minimum) n = 3 replicates. (**E**,**F)** CaCO2-control, CaCO2-BRAF^V600E^, CaCO2-KRAS^G12V^, HT29 and SW480 cells grown in physiological (1.0 g/L) glucose concentrations were treated with 10 µM Rapamycin and 10 µM OSI027 (+) or DMSO (−) for 24 h. Viable cells compared to DMSO are depicted. glc: glucose. Shown are standard deviation of (in minimum) n = 3 replicates. p < 0.05 was indicated with asterisk (unpaired two-tailed *t* Test).

Next we examined the viability (Tryphan blue staining) and apoptosis (Propidium iodide staining) of BRAF^V600E^- and KRAS^G12V^-expressing cells that had been provided different concentrations of glucose: none (0.0 g/L), low (0.3 g/L), physiological (1.0 g/L), intermediate (2.5 g/L) and high (4.5 g/L). CaCO2-BRAF^V600E^ and CaCO2-KRAS^G12V^ expressing cells showed a significant increase in viability at physiological glucose levels compared to CaCO2-control cells (Fig. 2A). Low levels of glucose and glucose starvation led to apoptosis in CaCO2-control and CaCO2-KRAS^G12V^ cells (Fig. 2A and Supplementary Figure 2A). A low supply of glucose diminished the viability of CaCO2-BRAF^V600E^ cells; however, viability was higher in the complete absence of glucose compared to 0.3 g/L glucose (Fig. 2A). A high supply of glucose (4.5 g/L) reduced cell viability in CaCO2-control CaCO2-BRAF^V600E^ cells, and to a significantly stronger extent in CaCO2-KRAS^G12V^ cells (Fig. 2A). Intermediate glucose levels led to the highest viability of CaCO2-control and CaCO2-BRAF^V600E^ cells, but not CaCO2-KRAS^G12V^ cells, which showed a narrowly optimal viability at 1.0 g/L glucose (Fig. 2A). In the colon cancer cell line HT29, which harbors an endogenously BRAF^V600E^ mutation, viability showed a dependency on glucose levels similar to CaCO2-BRAF^V600E^ (Fig. 2B, Supplementary Figure 2B). SW480 cells expressing endogenously mutant KRAS^G12V^ are optimally viable at the physiological 1.0 g/L glucose level. They undergo increased apoptosis in the absence of glucose, and at low, intermediate and high amounts of glucose. (Fig. 2B, Supplementary Figure 2B).

### BRAF^V600E^ and KRAS^G12V^ were sensitive for mTOR inhibition

Since glucose-dependent viability and apoptosis induction differed in cells expressing BRAF^V600E^ and KRAS^G12V^, we carried out the basal phosphorylation states of MEK, ERK and mTOR in BRAF^V600E^- and KRAS^G12V^-expressing cell lines cultivated with different glucose supplies using a bead-based phospho-proteomics approach (LUMINEX). MEK and ERK phosphorylation was increased under all glucose conditions in CaCO2-BRAF^V600E^ and CaCO2-KRAS^G12V^ cells compared to CaCO2-control cells (Fig. 2C). Interestingly, phospho-mTOR levels decreased in CaCO2-KRAS^G12V^ cells cultivated at intermediate and high glucose levels, a situation associated with the strong decrease in viability reported above. In contrast, we detected a slight raise of mTOR phosphorylation in CaCO2-BRAF^V600E^ and CaCO2-control cells at the same concentrations. Along with the decrease in cell growth we observed a drop in the phosphorylation of mTOR specifically at 0.3 g/L glucose in CaCO2-BRAF^V600E^. A similar regulation of phospho-mTOR levels was also evident in SW480 and HT29 cells (Fig. 2D).

The protein mTOR is a master switch for cellular metabolism that profoundly affects the regulation of viability and apoptosis^17^. We investigated mTOR’s impact on cell growth in BRAF^V600E^- and KRAS^G12V^-expressing cells under physiological glucose concentrations of 1.0 g/L while applying Rapamycin (10 µM) and a second small molecule that specifically inhibits both mTORC1 and mTORC2 (OSI027, 10 µM). These inhibitors decreased downstream phosphorylation of 4eBP1 and S6K, two protein target of mTOR (Supplementary Figure 2C–E), and significantly reduced viability in the cell lines CaCO2-BRAF^V600E^, and CaCO2-KRAS^G12V^ (Fig. 2E), and in HT29 and SW480 cells (Fig. 2F). Rapamycin, an mTORC1 inhibitor, promoted viability in CaCO2-control cells, however, reduced P-4EBP1 expression^18,19^; whereas the application of the mTOR inhibitor OSI027 reduced the growth of CaCO2-control cells (Fig. 2E). In contrast to targeting mTOR the inhibition of the mTOR upstream target AKT by MK-2206 did not alter the viability of CaCO2-BRAF^V600E^ and HT29 cells while it clearly increased the viability of KRAS^G12V^-expressing cells (Supplementary Figure 2F,G). Beside the blockade of phospho-AKT phosphorylation of 4eBP1 decreased only in CaCO2-control cells and remained unaffected in cells expressing BRAF^V600E^ and KRAS^G12V^ after the application of MK2206 AKT inhibitors (Supplementary Figure 2H-J).

Previous reports have suggested a direct interaction between BRAF and RAPTOR, a component of the mTOR signaling complex^20,21^. Therefore, we tested the interaction of BRAF and the mTORC1 complex through immunoprecipitation and subsequent Western blotting for BRAF and RAPTOR in CaCO2-control, CaCO2-BRAF^V600E^ and CaCO2-KRAS^G12V^ cells. BRAF^V600E^ binds stronger to RAPTOR compared to wildtype BRAF of CaCO2-control and CaCO2-KRAS^G12V^ cells which supports a potential effect onto mTOR signaling (Supplementary Figure 2K).

### BRAF^V600E^ induces a decoupling of mTOR from AMPK signaling

**Figure 3:**
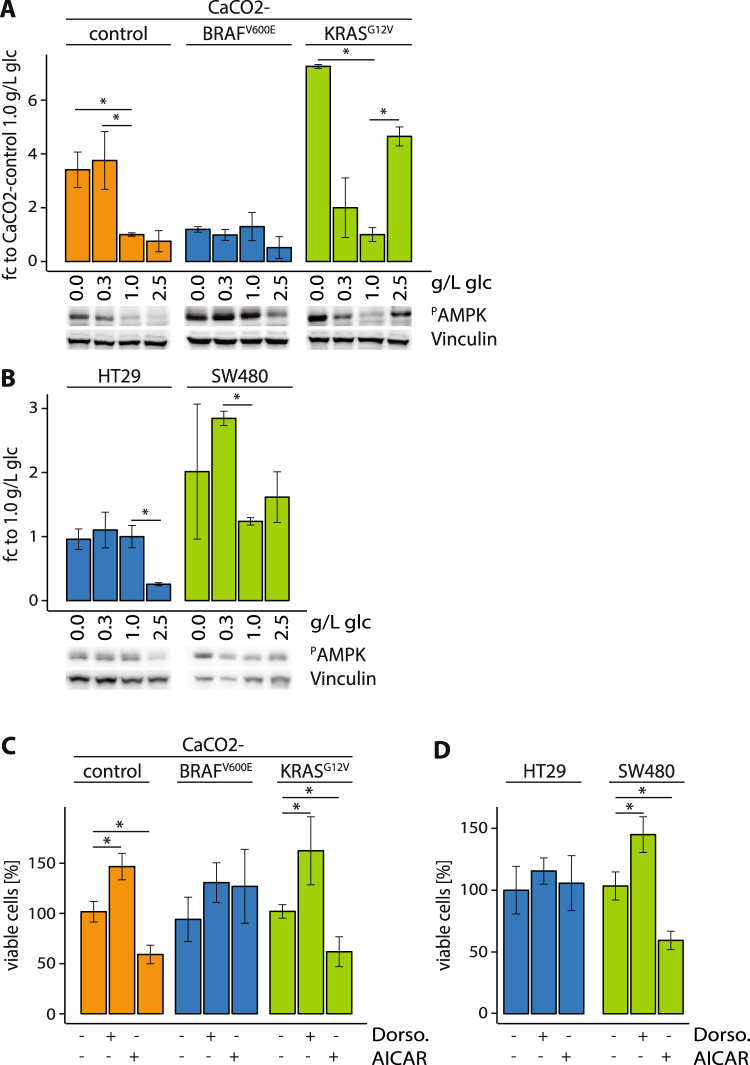
Decoupled AMPK signaling to mTOR in cells expressing BRAF^V600E^. (**A**,**B)** CaCO2-control, CaCO2-BRAF^V600E^, CaCO2-KRAS^G12V^, HT29 and SW480 cells cultivated with indicated glucose concentrations were analyzed with antibodies against phospho-AMPK (Thr172). Vinculin served as loading control. Shown are fold changes (fc) to 1.0 g/L glucose (previously normalized to Vinculin). For each cell line samples from different glucose concentrations were loaded together on one gel. Representative images are shown. Band intensities were measured using Image J. Western blot results were combined from (in minimum) n = 3 independent experiments. p < 0.05 was indicated with asterisk (unpaired two-tailed *t* Test). Full-length blots are shown in the Supplementary Information. (**C**,**D)** CaCO2-control, CaCO2-BRAF^V600E^, CaCO2-KRAS^G12V^, HT29 and SW480 cells grown with 1.0 g/L glucose were treated with 1 µM AMPK inhibitor Dorsomorphin (Dorso., +), 1 mM AMPK activator AICAR (+) or DMSO (−) for 24 h. Shown are viable cells compared to DMSO. glc: glucose. Shown are standard deviation of (in minimum) n = 3 replicates. p < 0.05 was indicated with asterisk (unpaired two-tailed *t* Test).

AMPK is a protein kinase that senses cellular energy states and acts upstream of mTOR. The AMPK axis plays a major role in suppressing tumor growth under conditions of energy stress, which led us to compare the glucose-dependent regulation of this pathway under BRAF^V600E^- or KRAS^G12V^-expressing conditions with that of control cells. The phosphorylation status of AMPK increased under low or no glucose concentrations in CaCO2-control and CaCO2-KRAS^G12V^ cells (Fig. 3A). Interestingly, phospho-AMPK levels also increased in KRAS^G12V^-expressing cells supplied with intermediate levels of glucose (2.5 g/L) correlating to the observed reduction of proliferation. The expression of BRAF^V600E^ induced no changes in phospho-AMPK levels after reduction of glucose (Fig. 3A). Again, similar patterns were observed in the cell lines with endogenously mutated KRAS and BRAF (Fig. 3B).

The application of the AMPK inhibitor Dorsomorphin (1 µM) increased the viability of CaCO2-control, and KRAS^G12V^-expressing cells, but had no effect on BRAF^V600E^-expressing cells (Fig. 3C and D). In these cell lines we observed the opposite effect after the application of AICAR (5-Aminoimidazole-4-carboxamide ribonucleotide, 1 mM), an activator of AMPK that mimics glucose deprivation. However, neither the inhibition nor activation of AMPK modulated cell growth in BRAF^V600E^-expressing cells, indicating that the AMPK/mTOR axis had been decoupled from nutrient deprivation.

### Metabolic characterization of BRAF^V600E^- and KRAS^G12V^-transformed cancer cells

#### Enhanced glycolytic flux in KRAS^G12V^-expressing cells

**Figure 4:**
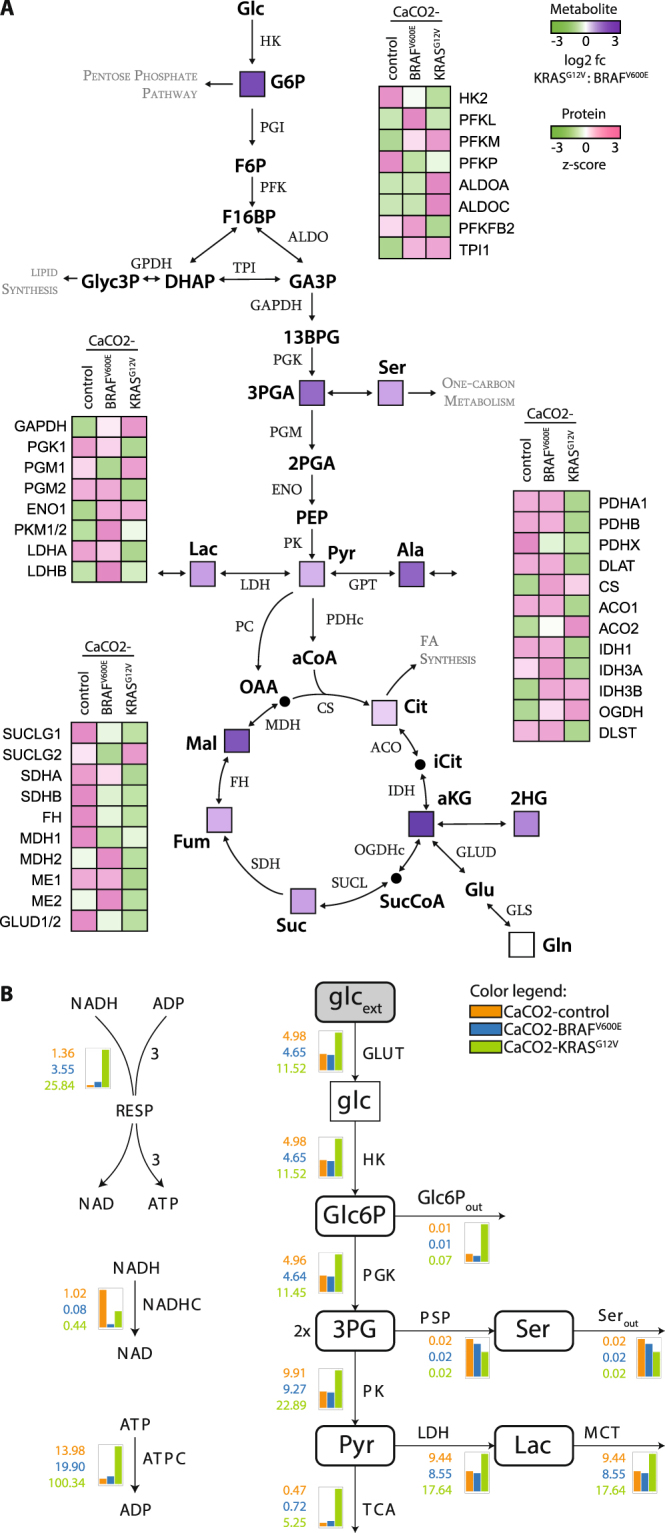
Cells expressing KRAS^G12V^ were highly glycolytic and accumulated lactic acid. (**A**) CaCO2-BRAF^V600E^ and CaCO2-KRAS^G12V^ cells were exposed to 1.0 g/L of ^13^C-glucose for 5 min, harvested and measured with GC-MS. For protein abundance, cells were measured with LC-MS. The mean ratio (log 2 fold changes, fc) of CaCO2-KRAS^G12V^ to CaCO2-BRAF^V600E^ for labeled metabolites and z-score of relative protein quantities are shown. (**B**) Mathematical model with the fluxes for each reaction. Species with rounded corners are species where concentration data was available. On the right side (or above) of edges are the reaction names, on the left side (or below) of the edges are the flux values of the reaction. The gray shaded node is an external species. Flux values are in µmol/s. 3PG: 3-phosphoglycerate, Glc6P: glucose-6-phosphate, HK: hexokinase, Lac: lactic acid, PGK: phosphoglyceratekinase, PSP: phosphoserine phosphatase, Pyr: pyruvic acid, Ser: serine. glc: glucose.

AMPK and mTOR links the regulation of viability to central carbon metabolism (CCM). CCM contributes to the generation of biosynthetic precursors and the maintenance of cellular energy homeostasis. Several reports suggest that the regulation of the CCM is directly controlled by the oncogenes RAF an RAS^8,14,22^. We performed an integrative analysis that combined shotgun proteomics and a time-resolved metabolomics (pSIRM) approach to illustrate oncogene-dependent alterations of CCM. The application of stable isotopes such as ^13^C-glucose in combination with gas chromatography-coupled mass spectrometry (GC-MS) provides a quantitative and carbon-resolved analysis of CCM. The temporal and pathway activity dependent incorporation of carbon-13 permits a monitoring of the fate of nutrients within the network^23,24^. A rigorous filtering of our shotgun proteomics  data revealed ~1 000 proteins present in all three CaCO2 cell lines (Supplementary Figure 3A). Analyses of protein and metabolite levels revealed that the glycolytic mode was enhanced in CaCO2-KRAS^G12V^ compared to CaCO2-BRAF^V600E^ cells at physiological glucose levels (Fig. 4A, Supplementary Table 1). Compared to CaCO2-control CaCO2-KRAS^G12V^ cells showed an enhanced glycolytic flux as well while CaCO2-BRAF^V600E^ cells unveil a lower glycolytic flux (Supplementary Figure 3B,C). Cells expressing KRAS^G12V^ compared to cells expressing BRAF^V600E^ respond with an enhanced glycolytic mode at intermediate (2.5 g/L) glucose levels (Supplementary Figure 3D). Metabolite levels indicate a significant increase in the extracellular pool of ^13^C-glucose-labeled lactic acid at 2.5 g/L glucose in cells expressing KRAS^G12V^ compared to CaCO2-control and BRAF^V600E^-expressing cells (Supplementary Figure 4E).

#### Mathematical modeling replicates the KRAS^G12V^-induced glycolytic phenotype and predicts critical transport capacity for lactic acid

In order to understand the metabolic differences we next tried to build up a mathematical model. The kinetic model (which was described in detail in the material and methods part) predicts the different accumulations and fluxes to lactic acid and the tricarboxylic acid cycle (TCA) cycle. The model depicts the corresponding fluxes (Fig. 4B). The figure shows that the flux in CaCO2-KRAS^G12V^ cells is nearly double that of CaCO2-control or CaCO2-BRAF^V600E^ cells. The reaction rate constants were fitted for CaCO2-control, CaCO2-BRAF^V600E^ and CaCO2-KRAS^G12V^ cells by using the same rate constants but changing concentrations of the enzyme, cofactor, and species according to the experimental data (the flux to α-ketoglutarate was taken as reference). For fitting, we used the metabolic time course data (^13^C-glucose) and shotgun proteomics (relative quantities) for physiological glucose conditions (1.0 g/L). The fluxes of lactic acid into the TCA cycle were validated by comparing them to the fluxes calculated from pSIRM metabolomics data.

**Figure 5:**
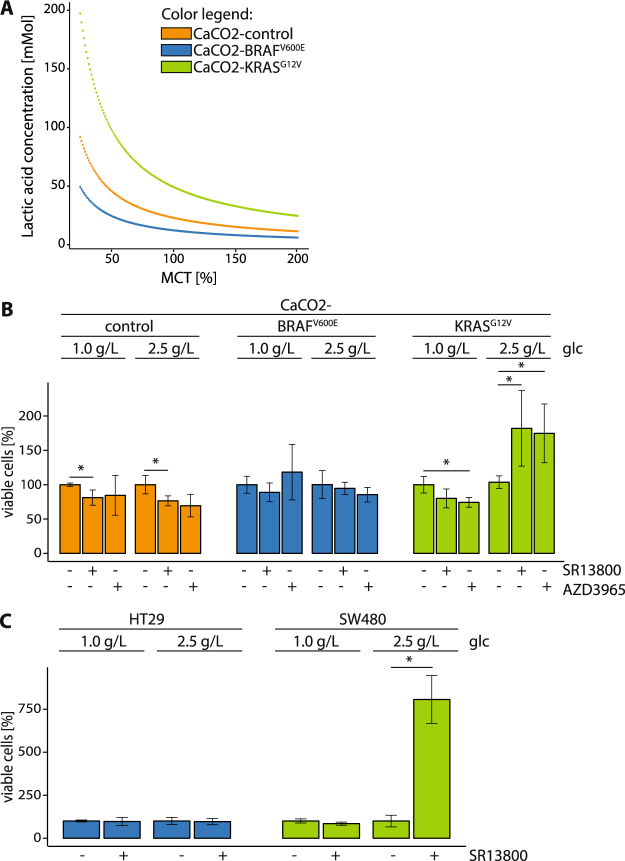
Cells expressing BRAF^V600E^ and KRAS^G12V^ are resistant to MCT1 inhibition. (**A**) Sensitivity analysis of lactic acid concentration depending on the MCT enzyme concentration. The MCT enzyme concentration varies here from 25% to 200% of the fitted value. The model shows how changing the MCT concentration affects the lactic acid accumulation for each cell line model. Low MCT enzyme concentrations result in increasingly large lactic acid concentrations. (**B**,**C**) CaCO2-control, CaCO2-BRAF^V600E^, CaCO2-KRAS^G12V^, HT29 and SW480 cells grown in physiological (1.0 g/L) and intermediate (2.5 g/L) glucose concentrations were treated with 0.1 µM MCT1 inhibitor SR13800 (+), 10 µM AZD3965 (+) or DMSO (−) for 24 h. Viable cells compared to DMSO are depicted. Shown are standard deviation of (in minimum) n = 3 replicates. p < 0.05 was indicated with asterisk (unpaired two-tailed *t* Test). glc: glucose.

Monocarboxylic acid transporters (MCTs) facilitate the shuttling of lactic acid across the plasma membrane. Glycolytic cells export lactic acid, while cells in tissues such as the brain or heart import lactic acid to fuel respiration^25^. This transport is proton-coupled and catalyzed by MCT1 and MCT4 [29]. Because our model predicted an accumulation of lactic acid in cells expressing KRAS^G12V^, we hypothesized that the capacity of the transporters might be limited. A sensitivity analysis based on the model suggested that MCT abundance would influence the accumulation of lactic acid in CaCO2-control, CaCO2-BRAF^V600E^ and CaCO2-KRAS^G12V^ cells (Fig. 5A). For the model, standard conditions (1.0 g/L glucose) were assumed in the CaCO2-control cells and represented as 100% MCT activity. Lowering the activity of MCT resulted in an accumulation of intracellular lactic acid, particularly in KRAS^G12V^-expressing cells, because it was being synthesized at high levels.

These results suggested that MCT1 inhibitors might have the same effect, because of a proposal that MCT1 inhibition selectively targets highly glycolytic cancer cells. We selected the small molecule inhibitor SR13800 (0.1 µM) to test this, given that it is a potent MCT1 inhibitor which has been shown to block the uptake of lactic acid in breast cancer cells *in vitro*^26^. We also applied AZD3965 (10 µM), another MCT1 inhibitor which is currently being used in a phase-I/II clinical trial as a therapy for advanced solid tumors (https://clinicaltrials.gov/ct2/show/NCT01791595). The application of SR13800 or AZD3965 *in vitro* induced a slight reduction of viability in CaCO2-control cells at physiological and intermediate glucose levels, while there was no change in the viability of cells expressing BRAF^V600E^ (Fig. 5B and C). The viability of CaCO2-KRAS^G12V^ cells at physiological glucose levels dropped after the inhibition of MCT1. Surprisingly, inhibiting MCT1 and raising the supply of glucose to 2.5 g/L increased these cells’ viability.

Previously published data suggest that MCT1 and MCT4 are functionally redundant^27^. A higher expression of MCT4 was found to be associated with resistance to AZD3965^28^. The determination of *MCT1* mRNA levels showed that their expression remained constant in CaCO2-control, CaCO2-BRAF^V600E^ and CaCO2-KRAS^G12V^ cells cultivated at physiological and intermediate glucose concentrations (Supplementary Figure 4A). Interestingly, we observed an oncogene-dependent expression of *MCT4* at physiological and intermediate levels of glucose. MCT4 expression was found higher in CaCo2 KRAS^G12V^ cells but did not show a glucose dependency. The application of MCT1 inhibitor SR13800 did not alter expression levels of *MCT1* or *MCT4* at physiological or intermediate glucose concentrations in any of the cell lines we analyzed (Supplementary Figure 4B,C).

#### Cells expressing KRAS^G12V^ are sensitive for glycolytic inhibition, while cells expressing BRAF^V600E^ show resistance

**Figure 6:**
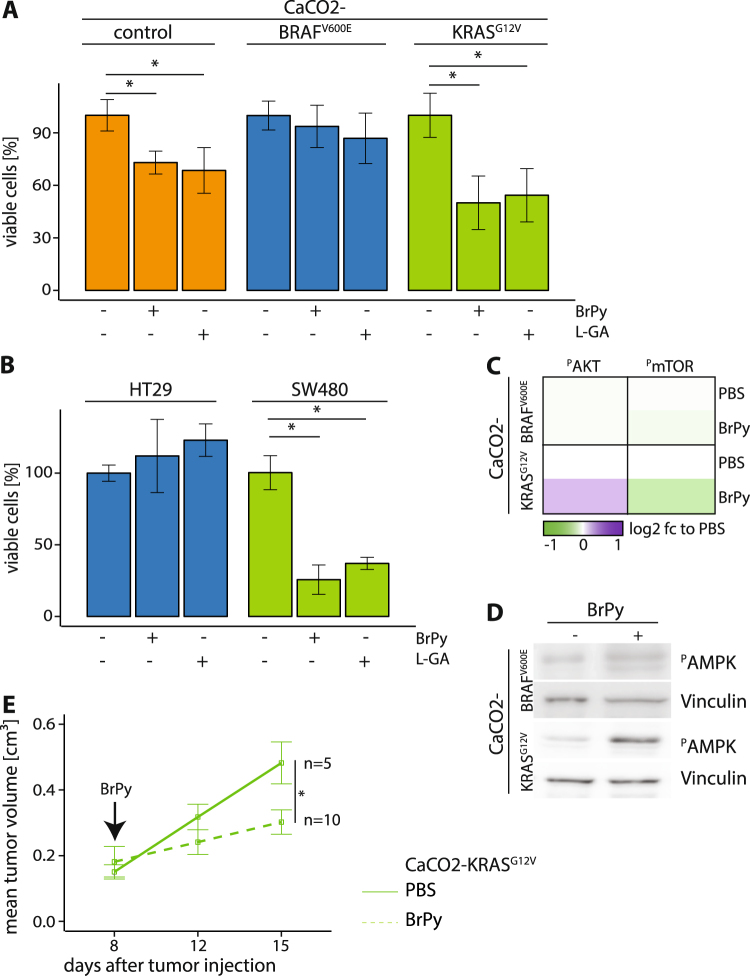
Cells expressing KRAS^G12V^ were sensitive for glycolytic inhibition *in vitro* and *in vivo*. (**A**,**B**) CaCO2-control, CaCO2-BRAF^V600E^, CaCO2-KRAS^G12V^, HT29 and SW480 cells were grown in physiological (1.0 g/L) glucose concentrations and treated with 0.25 mM 3-Bromopyruvate (BrPy), 1 mM L-glyceraldehyde (L-GA) or PBS (−) for 24 h. Viable cells compared to PBS are depicted. Shown are standard deviation of (in minimum) n = 3 replicates. p < 0.05 was indicated with asterisk (unpaired two-tailed *t* Test). (**C**) Phosphorylation of signaling molecules were analyzed with ELISA bead-based phosphoproteomics technology (Luminex). Shown are log2 fold changes (fc) to PBS. (**D**) CaCO2-BRAF^V600E^ and CaCO2-KRAS^G12V^ treated with 0.25 mM BrPy (+) or PBS (−) for 24 h were analyzed with antibodies against phospho-AMPK (Thr172). Vinculin served as loading control. Representative images are shown. Full-length blots are shown in the Supplementary Information. (**E**) CaCO2-KRAS^G12V^ cells were subcutaneously injected into the right flank of mice receiving 200 µL PBS or BrPy (8 mg/kg) in the presence of Doxycycline treatment every 2 days (starting from day 8) by intraperitoneal injection. Mean of tumor volume is shown for indicated mice per group up to 15 d. Shown are standard deviation of n = 5 (control group) and n = 10 (BrPy group) replicates. p < 0.05 was indicated with asterisk (unpaired two-tailed *t* Test).

Since we found that KRAS^G12V^-expressing cells were insensitive for MCT1 inhibition yet highly glycolytic, according to proteomic and metabolomic analyses combined with mathematical modelling, we suggest that they might have an increased sensitivity to the inhibition of glycolysis. As far back as the 1920s a number of studies demonstrated that the glycolysis of cancer cells could be inhibited by glycolytic inhibitors^24,29–31^. We assessed the viability of CaCO2-control, CaCO2-BRAF^V600E^ and CaCO2-KRAS^G12V^ cells treated with 0.25 mM 3-bromopyruvic acid (BrPy) or 1 mM L-glyceraldehyde (L-GA) (Fig. 6A). Each inhibitor reduced the growth of CaCO2-control and CaCO2-KRAS^G12V^ cells, but neither affected the viability of CaCO2-BRAF^V600E^ cells. In line with these results, SW480 cells were sensitive to glycolytic inhibition, while HT29 cells remained resistant (Fig. 6B). The phosphorylation of AKT and mTOR was unaltered in CaCO2-BRAF^V600E^ cells after treatment with BrPy (Fig. 6C), while cells expressing KRAS^G12V^ showed a downregulation of phospho-mTOR and an upregulation of phospho-AKT after glycolytic inhibition. To verify the glycolytic stress response after addition of BrPy the phosphorylation of AMPK was measured. Phospho-AMPK increased in both CaCO2-KRAS^G12V^ and CaCO2-BRAF^V600E^ expressing cells after inhibition of glycolysis (Fig. 6D).

We also tested the efficiency of the BrPy under *in vivo* conditions. CaCO2-control and CaCO2-BRAF^V600E^ cells injected into NSG mice that were receiving Doxycycline supplements did not develop tumors (Supplementary Figure 5A). Therefore only CaCO2-KRAS^G12V^ cells were used for BrPy treatment. We proceeded with the treatment using PBS (control) or BrPy (8 mg/kg) eight days following injection, a point at which tumors had reached 0.2 cm^3^. The average tumor volume was significantly reduced in BrPy-treated mice compared to the PBS control group after 15 days of treatment (Fig. 6E). A hematoxylin-eosin staining of tumor sections revealed extensive necrosis after the application of BrPy (Supplementary Figure 5B).

## Discussion

Since the discovery that BRAF and KRAS are oncogenic drivers of colorectal cancer, much progress has been made in understanding their common and individual effects^1^. However, targeting the RAF/ERK or PI3K/AKT pathways often results in drug resistance and therapy failure mediated by crosstalk and feedback mechanisms^4–6,32^. In addition, the search for inhibitors that block metabolism has been now in clinical evaluation stage, but most of the studies failed. This has created an urgent need for a better understanding of the molecular details associated with BRAF^V600E^- and KRAS^G12V^-driven cancers and their specific vulnerabilities. We used an integrated proteomics and metabolomics systems biology approach to investigate the impact of BRAF^V600E^ and KRAS^G12V^ on metabolism and signaling. We integrated our results into a mathematical model that could be used to predict the effects on various stages of metabolic processing within different cell types, in response to different levels of glucose, and compared the model to experimental results in which specific components of the RAF/ERK or PI3K/AKT were inhibited. We found an oncogene-specific regulation of nutrient-dependent signaling and glycolytic phenotypes that indicate specific, potential vulnerabilities in the pathways and the suppression of metabolic transport systems.

BRAF^V600E^ or KRAS^G12V^ alter cancer phenotypes through metabolic reprogramming. Hallmarks of tumor development are high glucose transport and glycolytic flux, which drive the production of ATP and intermediates necessary for anabolic processes. It has recently been shown that BRAF^V600E^ and KRAS^G12V^ are able to alter the systems by which they carry out glycolysis, phosphoserine biosynthesis, and glutamine metabolism and experience changes in the non-oxidative pentose phosphate pathway^8^. Our findings reveal that expressing KRAS^G12V^ is sufficient to highly drive glycolysis; however, at the same time this leads to a toxic production of intra-and extracellular lactic acid under specific levels of glucose supply. Further, the high glycolytic flux is predicted by our mathematical model, which integrates data from pSIRM metabolomics and shotgun proteomics experiments. The increased glycolytic flux promotes the excessive proliferation of cells and the activation of anti-apoptotic pathways. As tumorigenesis proceeds, KRAS^G12V^-expressing tumors become addicted to aerobic glycolysis and vulnerable to glucose deprivation. EGFR-independent signaling triggered by KRAS^G12V^ can increase the uptake of glucose by upregulation of GLUT1, which in turn enhances glycolytic activity and increases the production of lactic acid.

Glycolytic tumor cells often exhibit an overexpression of proton-linked monocarboxylate transporters (MCTs), which allow them to remove the large amounts of H^+^ ions produced by lactic acid production. The fact that MCT1 and MCT4 are commonly co-expressed in tumors suggests that MCT1 inhibition should have a therapeutic benefit in these tumors^26^. In line with this, the inhibition of MCT1 has been shown to inhibit highly glycolytic cancer cells^28^. Our mathematical model predicts that the transport capacity of MCT is critical for intracellular lactic acid levels, when we use levels of MCT as a surrogate parameter for the transport capacity of the cells. Nevertheless, pharmacological MCT1 inhibition has been inefficient in both oncogenic cell lines, which indicates that cells are able to engage some form of escape mechanism. Moreover, we found that intermediate levels of glucose in cells expressing KRAS^G12V^ can even stimulate their increased growth after MCT1 is inhibited. This indicates that MCT1 inhibition alone might cause unwanted adverse effects if it is used as the single therapeutic target in glycolytic tumors, depending on the nutrient supply.

In addition to its role in the transport of monocarboxylates, MCT1 is also necessary and sufficient for the uptake of BrPy by cancer cells. BrPy is a halogenated pyruvate derivative and strongly alkylates cysteine residues in proteins^33^. As an inhibitor of glycolysis, BrPy is known to have anti-tumorigenic functions^24^. We verified that tumor cell proliferation was reduced in the presence of KRAS^G12V^ after inhibition of glycolysis *in vitro* and *in vivo*. CaCO2-BRAF^V600E^ cells were not able to produce tumors when injected into NSG mice. However, *Sun et al*. used HT29 and SW480 cells harboring a naturally occurring BRAF^V600E^ or KRAS^G12V^ mutation for xenografts experiments, respectively and provided evidence for BrPy as an inhibitor of colon cancer growth *in vivo* and *in vitro*^34^. However, the authors were not able to decipher why this led to high levels of cell death in tumors expressing KRAS^G12V^, while those expressing BRAF^V600E^ were affected at much lower levels. We conclude that also in our xenograft experiments both a reduced proliferation and induction of apoptosis are present similar to the results of Sun and co-workers. Our finding that cells expressing KRAS^G12V^ are highly glycolytic and experience optimal growth at physiological glucose concentrations means that they are highly sensitive to glycolytic inhibition, and it creates a bottleneck for these tumors. Our study suggests that glycolytic inhibition may indeed be a suitable method of treating colorectal carcinomas expressing KRAS^G12V^.

Another issue to be resolved was how specific pathways respond to the BRAF and KRAS oncogenes. AKT is a proto-oncogene that mediates carcinogenesis and tumor progression by promoting cell survival and inhibiting apoptosis. AKT can also activate mTOR, a second key regulator of cell growth, by inhibiting TSC2 or blocking PRAS40^35^. AKT and mTOR induce glycolysis by increasing the trafficking of GLUT1 and the activation of glycolytic enzymes such as phosphofructokinase and hexokinase^36^. In non-cancerous cells, mTOR is activated under high levels of glucose and blocked by low amounts of nutrients. The AMPK-mediated inactivation of mTORC1 is a major metabolic checkpoint^37^. We discovered that KRAS and BRAF-dependent signaling via MEK and were similar even under varying amounts of glucose. However, signaling to mTOR differs between mutant KRAS and BRAF, and this results in distinct phenotypes and metabolic cues.

**Figure 7:**
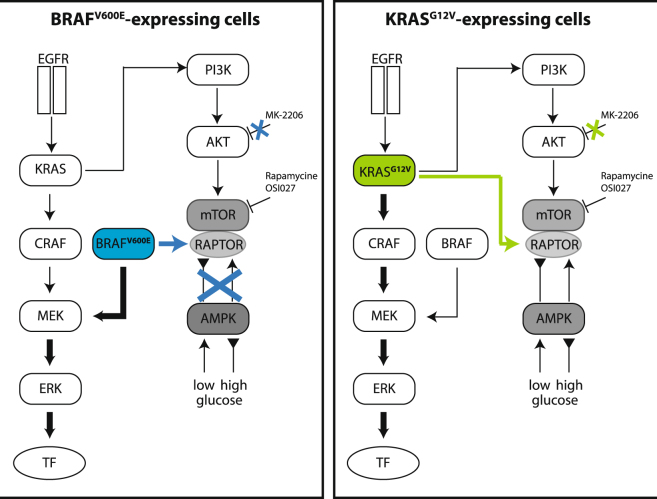
Signaling towards mTOR differs in cells expressing KRAS^G12V^ and BRAF^V600E^. Mitogenic signaling via EGFR did not differ in cells expressing BRAF^V600E^ and KRAS^G12V^. Cells expressing BRAF^V600E^ showed a disruption of AMPK/mTOR signaling leading to inhibitor resistance. In cells expressing KRAS^G12V^, AKT/mTOR signaling is disrupted resulting in inhibitor resistance; however signaling via AMPK is functional.

It is known that MAPK pathways and mutations within it are involved in the regulation of cellular processes such as proliferation and apoptosis. Because these responses are also regulated by cellular energy levels, signaling between AMPK, mTOR, AKT and MAPKs is linked in a network. Cells expressing BRAF^V600E^ and KRAS^G12V^ exhibit a disconnection of AKT signaling towards mTOR, as observed by activated downstream signaling leading to enhanced viability after inhibition of AKT. Further, a biomarker-driven phase 2 study of MK-2206 AKT inhibitor in patients with colorectal cancer showed no clinical efficiency and the desired level of target inhibition could not be achieved^38^. Our data revealed that any attempt to inhibit mTOR upstream will likely be unsatisfactory in cells expressing BRAF^V600E^ or KRAS^G12V^ (Fig. 7).

By applying different levels of glucose, we showed that cells expressing KRAS^G12V^ experience optimal growth when cultivated under physiological levels. A rise or decline in glucose conditions results in an AMPK-mediated downregulation of viability and the induction of apoptosis. We found that changing glucose levels did not affect colorectal cancer cells expressing BRAF^V600E^ because of the decoupling of AMPK from mTOR signaling (Fig. 7).

Melanoma cells expressing BRAF^V600E^ have a very limited response to metabolic stress due to the constitutive dissociation of the LKB1-AMPK complex in these cells^39^. They proliferate even under low glucose conditions when LKB1 and AMPK are disconnected. AMPK is probably decoupled from mTOR signaling in cells expressing BRAF^V600E^ because it binds to RAPTOR, as well as to Rheb, which has been previously shown^20,40^. mTORC1 activity is suppressed by low glucose amounts only when AMPK phosphorylates its substrate RAPTOR. This phosphorylation is also necessary for AMPK’s functions as a metabolic checkpoint.

We suggest that since AMPK is deregulated in BRAF^V600E^-expressing cells, they are driven by a different resistance mechanism involving altered apoptotic- or autophagy-inducing programs. Both BRAF^V600E^- and KRAS^G12V^-expressing cells were sensitive to mTOR inhibition, since Rapamycin disrupts the interaction between RAPTOR and mTOR^41^. Evidence of the further importance of mTOR or its downstream targets has come from studies showing that OSI027, which binds to the catalytic site of mTOR and blocks its kinase function, inhibits tumor growth and down regulates phospo-4eBP1 in xenografts models. The reduced tumor volume after treatment with mTOR inhibitors was already shown in in GEO (KRAS^G12A^; both OSI027 and Rapamycin), SW480 (KRAS^G12V^; Rapamycin), LS174T (KRAS^G12D^; Rapamycin) and HT29 (BRAF^V600E^) xenografts^42–44^. *Bhagwat and colleagues*^21^ demonstrated the dose-dependent efficiency of OSI027 in MDA-MB-231 (KRAS^G13D^, BRAF^G464V^) xenografts and a reduced tumor volume after treatment with OSI027 and Rapamycin in GEO (KRAS^G12A^), COLO205 (BRAF^V600E^) and ovarial carcinoma (SKOV-3, OVCAR-5, IGR_OV1) xenografts. The phosphorylation of mTOR substrate 4eBP1 was decreased in cells expressing BRAF^V600E^ and KRAS^G12V^ after mTOR inhibition, but not through a disruption in the pathway upstream of mTOR.

In summary our data revealed that colorectal cancer cell lines expressing BRAF^V600E^ and KRAS^G12V^ are similar in terms of their morphologies and mitogenic signaling, but exhibit fundamental differences in mTOR-mediated signaling. KRAS^G12V^ induces a glycolytic dependency, while BRAF^V600E^ acquires metabolic stress resistance by decoupling AMPK from mTOR signaling. But interestingly, mTOR inhibition counteracted the metabolic phenotype. This suggests that mTOR should be considered as a potential therapeutic target in BRAF^V600E^-driven colorectal cancer.

## Material and Methods

### Cells and cell culture

The cell lines SW480 and HT29 were obtained from ATCC (American Type Culture Collection, Teddington, United Kingdom). The cell lines were maintained in DMEM (Dulbeccos Modified Eagles Medium, Thermo Fischer Scientific, Waltham, MA, USA) supplemented with 10% fetal calf serum (FCS, Thermo Fischer Scientific, Waltham, MA, USA), 1% penicillin/streptomycin (Thermo Fischer Scientific, Waltham, MA, USA) and 1 g/L glucose (Sigma-Aldrich, St. Louis, MO, USA). CaCO2-empty vector (CaCO2-control), CaCO2-BRF^WT^, CaCO2-BRAF^V600E^ and CaCO2-KRAS^G12V^ cells have been described previously and were a kind gift of Tilman Brummer (Institute of Molecular Medicine and Cell Research, Centre of Biochemistry and Molecular Cell Research (ZBMZ), Freiburg, Germany). The Doxycycline inducible expression system is described in detail elsewhere^4^. The cells were maintained in glucose-free, glutamine-free and phenolred-free DMEM (Dulbeccos Modified Eagles Medium, Thermo Fischer Scientific, Waltham, MA, USA) supplemented with 10% FCS (Thermo Fischer Scientific, Waltham, MA, USA), 1% penicillin/streptomycin ((Thermo Fischer Scientific, Waltham, MA, USA), 5 µg/mL puromycine (Calbiochem, Billerica, MA, USA), 5 µg/mL blasticidine (AppliChem, Darmstadt, Germany), Doxycycline (2 µg/mL, Sigma-Aldrich, St. Louis, MO, USA), 4 mM glutamine (Thermo Fischer Scientific, Waltham, MA, USA) and 1 g/L glucose (Sigma-Aldrich, St. Louis, MO, USA). All cells were incubated in a humidified atmosphere of 5% CO_2_ in air at 37 °C. For morphology studies, cells were cultured w/o Doxycycline, plated and photographed after 24 h (starting day). Shortly after Doxycycline induction indicated glucose concentrations were added for up to 16 d. Pictures were captured with Zeiss Axio Observer Z1 microscope. One representative picture is shown. For all other experiments depicted concentrations of glucose were added for 3 d. Cells were plated with indicated glucose amount and analyzed after 48 h. For inhibitor studies, 24 h after plating inhibitor was added and cells were counted after additional 24 h. Cells were counted with TC20 cell counter (BioRad, Hercules, CA, USA) using Trypan blue for detection of viable cells.

### Reagents

For inhibitor studies the following reagent were used: The glycolysis inhibitors 3-bromopyruvate (Sigma-Aldrich, St. Louis, MO, USA) and L-glyceraldehyde (Sigma-Aldrich, St. Louis, MO, USA) solved in PBS were added from a sterile stock solution to final concentration of 0.25 mM and 1 mM, respectively. SR13800 (Tocris, Bristol, United Kingdom) and AZD3965 (Selleck Chemicals, Houston, TX, USA) - inhibitors of MCT1 -solved in DMSO were added from a sterile stock solution to final concentration of 0.1 µM and 10 µM, respectively. AKT inhibitor MK-2206 (Selleck Chemicals, Houston, TX, USA) solved in DMSO were added from a sterile stock solution to final concentration of 1 µM. mTOR inhibitors Rapamycin (Calbiochem, Billerica, MA, USA) and OSI027 (Selleck Chemicals, Houston, TX, USA) solved in DMSO were added from a sterile stock solution to final concentration of 10 µM. AMPK inhibitor Dorsomorphin and AMPK activator AICAR (both Selleck Chemicals, Houston, TX, USA) solved in DMSO were added from a sterile stock solution to final concentration of 1 µM or 1 mM, respectively. As control, inhibitor solvent was used.

### RNA isolation and quantitative RT-PCR analysis

RNA was isolated using the RNeasy-mini-kit (Qiagen, Hilden, Germany) according to the supplier’s protocol. To obtain cDNA from RNA, the high-capacity cDNA reverse transcription kit (Applied Biosystems, Foster City, CA, USA) was used. Synthesis of double-stranded DNA during the PCR cycles was visualized with TaqMan gene expression assays FAM-dye labeled (gene of interest: *MCT1* Hs01560299 m1, *MCT4* Hs00358829 m1) or VIC-dye labeled for loading control *PGK1* (Hs 943178 g1) and TaqMan gene expression master mix (Applied Biosystems, Foster City, CA, USA). Quantitative real time PCR (qrtPCR) analysis was performed using a StepOne 96 well format Light Cycler apparatus (Applied Biosystems, Foster City, CA, USA). Experiments were run and analyzed with the StepOne 2.0 software. The data were analyzed quantitatively by measuring the threshold cycles (CT). CT values where normalized first by the CT of the internal control PGK1 (minus deltaCT) and second by the deltaCT value of the control (minus ddCT), which are interpreted as log2 fold changes.

### Luminex bead-based technology

Cell lysates were collected and the level of phospho-protein expression was analyzed with Luminex protein array system (BioRad, Hercules, CA, USA) using beads specific for phospho-MEK1 (S217/S221), phospho-AKT (S473), phospho-mTOR (S2448), phosphor-S6K (S235/S236) and phospho-ERK1/2 (Thr202/Tyr204, Thr185/Tyr187) according to the manufacturer’s instructions. Briefly, cells were washed with PBS and lysed with cell lysis buffer (BioRad, Hercules, CA, USA). Lysate protein concentration was determined with BCA (bicinchoninic acid) method (Thermo Fischer Scientific, Waltham, MA, USA). The beads and detection antibodies were diluted in a ratio of 1:3. For acquiring data, the BioPlex Manager software was used.

### Immunoblot

Protein extracts of cells were prepared by incubation with cell extraction buffer (Invitrogen, Carlsbad, CA, USA). Reagents for SDS-polyacrylamide gel electrophoresis (PAGE) and Western blotting were obtained from Bio-Rad Laboratories (Hercules, CA, USA) and Carl Roth (Karlsruhe, Germany). Electrophoresis was performed and the proteins were transferred onto nitrocellulose or PVDF membranes (polyvinylidene difluoride). Unbound protein sites were blocked with BSA (bovine serum albumin) or milk powder in phosphate-buffered saline (PBS) containing 0.1% Tween20. Thereafter, specific proteins were detected by incubation with primary antibodies overnight at 4 °C followed by specific secondary antibodies. Membranes were developed using ECL solution from GE Healthcare Life Science (Buckinghamshire, United Kingdom) and scanned with Image Quant LRS-2400 (Healthcare Life Science, Buckinghamshire, United Kingdom). Signals were quantified with ImageJ. The following antibodies were used: mouse anti-human BRAF clone F7 (1:500 Santa Cruz, Dallas, TX, USA), mouse anti-human panRAS clone RAS 10 (1:500 Millipore, Darmstadt, Germany), rabbit anti-human E-Cadherin clone H-108 (1:500 Santa Cruz, Dallas, TX, USA), rabbit anti human-phospho-LKB1 Ser428 clone C67A3 (1:500 New England Biolabs, Frankfurt a. M., Germany), rabbit anti-human phospho-AMPK Thr172 (1:500 New England Biolabs, Frankfurt a. M., Germany), rabbit anti-human phospho-4eBP1 Thr70 (1:500 New England Biolabs, Frankfurt a. M., Germany), rabbit anti-human Raptor 24C12 (1:500 new England Biolabs, Frankfurt a. M., Germany), rabbit anti-human ß-actin (1:2 000 New England Biolabs, Frankfurt a. M., Germany), mouse anti-human vinculin (1:2 000 Sigma-Aldrich, St. Louis, MO, USA), anti-rabbit IgG HRP (1:2 000 New England Biolabs, Frankfurt a. M., Germany) and anti-mouse IgG HRP (1:2 000 New England Biolabs, Frankfurt a. M., Germany). One representative picture is shown.

### Immunoprecipitation

Immunoprecipitation was performed using Dynabeads Protein G (Thermo Fisher Scientific, Waltham, MA, USA) according to manufacturer’s protocol. Pellets were lysed in cell lysis buffer (Thermo Fisher Scientific, Waltham, MA, USA) and BRAF was immunoprecipitated with 8 µg BRAF F-7 and detected by using BRAF and Raptor specific antibodies.

### Immunohistochemical staining

For detection of Mucin slides were stained with periodic acid Schiffs-reaction was performed (specific for neutral Mucin and glycoproteins). Slides were fixed in 10% formaline in PBS for 10 min following 1% period acid for 10 min. Schiffs reagent was added for 15 min following 3 min hematoxylin before covering. Alcian Blue (specific for acid Mucins) was added for 1 h. Sections were counter stained with hematoxylin to facilitate orientation. Images were captured with Leica microscope. Tumors were stored in 4% formalin solution, embedded in paraffin, and xenograft sections were stained with eosin and hematoxylin (HE) to facilitate cell types. One representative picture is shown.

### Flow cytometry of death cells

Cells were plated with indicated glucose concentration and trypsinized after 5 days. The resuspended cells were fixed with 10% formaldehyde and permeabilized using ice cold 90% methanol following staining with rabbit anti-human cleaved Caspase 3 antibody Alexa Fluor 647 (1:50 New England Biolabs, Frankfurt a. M., Germany). Cells were analyzed using Accuri C6 Flow Cytometer (BD company) and FlowJo 7.6.5 software.

### MUC5AC ELISA

MUC5AC was determined using ELISA Kit (Cloude-Clone, Houston, TX, USA) according to manufacturer’s instructions. The OD was measured at 450 nm using the plate reader Tecan Infinite 200 Pro (Männedorf, Swiss).

### Proteomics measurement

#### Cell harvest and sample preparation

Cell pellets from CaCO2-control, CaCO2-BRAF^V600E^ and CaCO2-KRAS^G12V^ cells cultivated with 0.0 g/L, 0.3 g/L, 1.0 g/L or 2.5 g/L glucose were resuspended in Urea containing buffer (8 M Urea, 100 mM TrisHCl, pH 8.25). After removing cell debris by centrifugation protein concentration was determined by BCA and 500 µg of protein for each sample were taken. Disulfide bridges of proteins were reduced in DTT 2 mM for 30 min at 25 °C and successively free cysteines alkylated in iodoacetamide 11 mM for 20 min at room temperature in the dark. Afterwards, LysC digestion was performed by adding 5 µg of LysC to the sample and incubated it for 18 h under gentle shaking at 30 °C. After LysC digestion, the samples were diluted 3 times with 50 mM ammonium bicarbonate solution, 7 µl of immobilized trypsin (Applied Biosystems, Foster City, CA, USA) were added and samples were incubated 4 h under ration at 30 °C. Digestion was stopped by acidification (pH 2) with trifluoroacetic acid and an 18 µg aliquot of peptide digest was desalted on StageTip^45^.

#### LC-MS/MS measurement and analysis

For all samples, 5 µL were injected in duplicates in a LC-MS/MS system (NanoLC 1D Plus [Eksigent, Dublin, CA, USA] coupled to LTQ-Orbitrap Velos [Thermo Fischer Scientific, Waltham, MA, USA] or QExactive Plus [Thermo Fischer Scientific, Waltham, MA, USA]), using a 240 min gradient ranging from 5% to 45% of solvent B (80% acetonitrile, 0.1% formic acid; solvent A = 5% acetonitrile, 0.1% formic acid). For the chromatographic separation 30 cm long capillary (75 pm inner diameter) was packed with 1.8 micron C18 beads (Reprosil-AQ, Dr. Maisch GmbH, Ammerbuch, Germany). On one end of the capillary nanospray tip was generated using a laser puller (P-2000 Laser Based Micropipette Puller, Sutter Instruments, Novato, CA, USA), allowing fretless packing.

The nanospray source was operated with a spray voltage of 2.1 kV and an ion transfer tube temperature of 260 °C. Data were acquired in data dependent mode, with a top20 method on the LTQ-Orbitrap Velos (one survey MS scan in the Orbitrap mass analyzer, 60 000 resolution at 400 m/z, followed by up to 20 MS/MS scans in the ion trap on the most intense ions, intensity threshold = 750 counts) or a top10 method on the QExactive Plus (one survey MS scan with resolution 70 000 at m/z 200, followed by up to 10 MS/MS scans on the most intense ions, intensity threshold 5 000). Once selected for fragmentation, ions were excluded from further selection for 30 sec, in order to increase new sequencing events.

Raw data were analyzed using the MaxQuant proteomics pipeline (v1.4.1.2) and built in the Andromeda search engine^46,47^. The IPI human database v3.71 was employed. Carbamidomethylation of cysteines was chosen as fixed modification, oxidation of methionine and acetylation of N-terminus were chosen as variable modifications. The search engine peptide assignments were filtered at 1% FDR; other parameters were left as default. Quality control was done as written in^48^. After removing reverse and contaminant 3 395 identifiers were found. For further data analysis Perseus (version 1.4.0.20^49^) was used. Data visualization was done using VANTED (Visualization and Analysis of Networks containing Experimental Data) 2.2.1 software^50^.

### Metabolomics measurement

#### Labeling and harvest

Cells were seeded in appropriate density after cultivation for 3 d in indicated glucose conditions. For labeling experiments, 24 h and 4 h before harvest media was changed to maintain high glycolytic activity. Media samples were collected 4 h and directly before labeling to analyze extracellular metabolites. ^13^C-glucose or ^13^C-glutamine (4 mM) was added for 2 min, 5 min or 8 min and 5 min, 15 min, 45 min or 60 min, respectively, while ^12^C-glucose was added for 5 min representing natural mass isotopic distribution. For ^13^C-glucose different glucose amounts were used (0.0 g/L, 0.3 g/L, 1.0 g/L, 2.5 g/L). Thereafter cells were shortly washed (20 sec) with a wash buffer containing labeled or non-labeled glucose and glutamine. Directly after washing cells were quenched by ice-cold methanol (50%) containing 2 µg/mL cinnamic acid.

#### Metabolite extraction, GC-MS measurement and analysis

Sampling, preparation, measurement and data analysis follows the description published in *Pietzke et al*.^23,24^. Shortly, dried cell extracts were dissolved in 10 µL of methoxyamine hydrochloride solution (40 mg/mL in pyridine) and incubated for 60 min at 30 °C with constant shaking followed by the addition of 25 µL of N-methyl-N-[trimethylsilyl]trifluoroacetamide (MSTFA) and incubation at 37 °C for 90 min. The extracts were centrifuged for 10 min at 10,000 × g, and aliquots of 15 µL were transferred into glass vials for gas chromatography-mass spectrometry (GC-MS) measurement. Metabolite analysis was performed on a Pegasus III-TOF-MS-System (LECO Corporation, St. Joseph, MN, USA) complemented with an auto-sampler (Gerstel). The samples were injected in split mode (split 1:5, injection volume 1 µL) in a temperature-controlled injector (CAS4, Gerstel, Mühlheim an der Ruhr, Germany) with a baffled glass liner. The following temperature program was applied during sample injection: initial temperature of 80 °C for 30 sec followed by a ramp with 12 °C/min to 120 °C and a second ramp with 7 °C/min to 300 °C and final hold for 2 min. Gas chromatographic separation was performed on an Agilent 6890 N (Agilent technologies, Santa Clara, CA, USA), equipped with a VF-5 ms column of 30-m length, 250-µm inner diameter, and 0.25-µm film thickness. Helium was used as carrier gas with a flow rate of 1.2 mL/min. Gas chromatography was performed with the following temperature gradient: 2 min heating at 70 °C, first temperature gradient with 5 °C/min up to 120 °C and hold for 30 s; subsequently, a second temperature increase of 7 °C/min up to 210 °C, and a third ramp of 12 °C/min up to 350 °C with a hold time of 2 min. The spectra were recorded in a mass range of 60 to 600 U with 20 spectra/s. The GC-MS chromatograms were processed with the ChromaTOF software (LECO Corporation, St. Joseph, MN, USA). Mass spectra data were extracted using the software tool Maui-VIA^51^. Acquired data were normalized to the internal standard cinnamic acid and cell count (1e^+6^ cells).

#### Colorectal carcinoma xenografts

CaCO2-control, CaCO2-BRAF^V600E^ and CaCO2-KRAS^G12V^ xenografts were grown by subcutaneous injection of 3 million cells in 0.2 mL of 1:1 serum-free RPMI:Matrigel into the mid-dorsal flank of male NSG mice. The mice were feed with Doxycycline to keep oncogene expression ongoing. For inhibitor studies, CaCO2-KRAS^G12V^ mice were randomized into BrPy (8 mg/kg) and PBS negative control group. Tumor size was measured twice a week using calipers. At sacrifice tumors were collected in 4% formalin solution or liquid nitrogen. All xenograft studies were performed by EPO (Experimental Pharmacology and Oncology) Berlin-Buch GmbH, Robert-Roessle-Str. 10, 13125 Berlin. EPO strictly follows the EU guideline European Convention for the Protection of Vertebrate Animals Used for Experimental and Other Scientific Purposes (EST 123) and the German Animal Welfare Act (revised version Art. 3 G v. 28.7.2014 I 1308). Furthermore, we handle our animals according to the Regulation on the Protection of Animals Used for Experimental or for Other Scientific Purposes (Tierschutz-Versuchstierverordnung- TierSchVersV: revised version Art. 6 V v. 12.12.2013 I 4145). The animal experiments were performed according to the German Animal Protection Law and with approval from the responsible local authorities (LaGeSo [Landesamt für Gesundheit und Soziales] Berlin, Germany). The *in vivo* procedures were consistent and in compliance with the UKCCCR (United Kingdom Coordinating Committee on Cancer Research) guidelines.

#### Mathematical model

We built a minimal kinetic model of glycolysis based on a published glycolysis model^52^ using irreversible mass action kinetics of the form

1 $$v=k\cdot E\cdot \prod _{i=1}^{n}ai$$

where *v* is the flux, *k* is the rate constant, *E* is the enzyme concentration, *n* is the number of substrates, and *ai* is the *i*th substrate concentration of the reaction. The metabolomics and proteomics data values were converted to µMol for the use in the model. For all species except glucose we had concentration measurements from the metabolomics data, so we set the values of species concentration to the median value of all data points (independent of time) for each species. For glucose, we used for CaCO2-control, CaCO2-BRAF^V600E^ and CaCO2-KRAS^G12V^ cells the default value 0.54. The external glucose concentration was calculated from knowing the glucose concentration in the medium and it was set constant. Cofactors were set to default values for CaCO2-control cells^53–55^ while in CaCO2-BRAF^V600E^ and CaCO2-KRAS^G12V^ cells cofactors were fitted.

We used the values from the (to µmol converted) proteomics data as follows:

PSP, PK, and LDH concentration values were taken directly.

GLUT, HK, and G6P_OUT_ were taken directly for CaCO2-control, for CaCO2-BRAF^V600E^ and CaCO2-KRAS^G12V^ cells they were fitted.

PGK and SEROUT were set to 0.1 for CaCO2-control, for CaCO2-BRAF^V600E^ and CaCO2-KRAS^G12V^ cells they were fitted.

ATPC, NADHC, and RESP were fitted for all CaCO2 cell lines.

MCT and TCA were set to 0.1 for CaCO2-control, for CaCO2-BRAF^V600E^ and CaCO2-KRAS^G12V^ cells they were fitted.

For parameter fitting, we used the Matlab toolbox D2D^56^ with multistart and steady-state constraints. First, we had to calculate the fluxes going into lactic acid and into the TCA cycle from the pSIRM metabolomics data to have reference values for the model. For the TCA cycle we used α-ketogluturate as the reference, because its flux was the highest. The calculation of the flux was done by equation 2.

2 $${v}_{data,i}=\frac{C13Poo{l}_{i}-C13Poo{l}_{t=0}}{{t}_{i}}$$

We had the percentage of ^13^C labeled molecules of the full pool for a species at each time point. Based on these values we calculated the absolute values of the ^13^C-labeled quantities for a species by multiplying the percentage of the ^13^C labeled molecules with the median value of all data points for the full pool of that species to get the ^13^C*Pool*_*i*_ values. To get the fluxes *vdata*,*i* we subtracted the initial pool of ^13^C labeled molecules (^13^C*Pool*_*t=0*_) and then divided by the time. The fluxes calculated from the data for lactic acid and the TCA cycle are our reference values to fit our models. We fitted all the *k* values of our kinetics (see eq. 1) for CaCO2-control and forced the fluxes to lactic acid and TCA cycle to be at least as high as the calculated fluxes suggested. After reaching a steady-state for the CaCO2-control model, we started fitting the other two cell lines. Thereby, we used the kinetic constants of CaCO2-control, changed the species concentration and enzyme concentration according to the cell line. The fitted value for GLUT slightly deviates from the proteomics data for CaCO2-BRAF^V600E^ cells, for CaCO2-KRAS^G12V^ cells we had not had a value for one of the isoenzymes in the first place, but the fitted value seems reasonable in the context of the values for the other two cell lines. In the case of HK and G6P_OUT_ the values of CaCO2-BRAF^V600E^ and CaCO2-KRAS^G12V^ cells were negligibly different, especially in consideration that we had not had any measurement values of glucose, and the Glc6P concentration was very low with some variance.

The values of MCT and TCA for CaCO2-BRAF^V600E^ and CaCO2-KRAS^G12V^ cells were fitted so that they are at least as high as the flux calculated from data. The reason for “at least as high” is that even though only ^13^C labeled molecules enter the system, there are still non ^13^C labeled molecules left that take part in reactions. Cofactors were fitted to be in the same magnitude as the cofactors of CaCO2-control.

After finishing the parameter fitting, we performed a sensitivity analysis to see the influence of the MCT enzyme concentration on the lactic acid accumulation for our models. As our kinetics are all irreversible, this could easily be done analytically.

Let us call the flux into lactic acid *v*_*in*_:

3 $${v}_{in}={k}_{LDH}\cdot LD{H}_{e}\cdot NADH\cdot Pyr$$

where *k*_*LDH*_ is the constant for the reaction rate, *LDH*_*e*_ is the LDH enzyme concentration, NADH is the NADH concentration and Pyr is the pyruvic acid concentration. *v*_*in*_ is constant for our analysis as it is not influenced by MCT. The differential equation for lactic acid has the form:

4 $$\frac{dLac}{dt}={v}_{in}-{k}_{MCT}\cdot MC{T}_{e}\cdot Lac$$

where *k*_*MCT*_ is the constant for the reaction rate, *MCT*_*e*_ the MCT enzyme concentration and Lac is the lactic acid concentration. Equation 4 combined with the knowledge that our system is in steady state gives:

5 $$\frac{dLac}{dt}=0\Rightarrow Lac=\frac{{v}_{in}}{MC{T}_{e}\cdot {k}_{MCT}}$$

Applying now the equation for the sensitivity analysis on lactic acid gives:

6 $$\frac{dLac}{dMC{T}_{e}}=-\frac{{v}_{in}}{MC{T}_{e}^{2}\cdot k}$$

The lower the *MCT*_*e*_ concentration is, the bigger is the lactic acid accumulation. Note that the MCT concentration, *k*_*MCT*_, and the *v*_*in*_ are different for each cell line.

### Statistical analysis

The unpaired Student’s t-test was used for statistical analysis. Results are presented as means of (in minimum) n = 2 replicates. A value of p < 0.05 was selected as the level of significance and indicated with asterisks.

## Electronic supplementary material

Supplementary Table and Figures

## References

[1] Morkel M, Riemer P, Blaker H, Sers C (2015). Similar but different: distinct roles for KRAS and BRAF oncogenes in colorectal cancer development and therapy resistance. Oncotarget.

[2] Dempke WC, Heinemann V (2010). Ras mutational status is a biomarker for resistance to EGFR inhibitors in colorectal carcinoma. Anticancer research.

[3] Temraz S, Mukherji D, Shamseddine A (2015). Dual Inhibition of MEK and PI3K Pathway in KRAS and BRAF Mutated Colorectal Cancers. International journal of molecular sciences.

[4] Fritsche-Guenther R (2016). Effects of RAF inhibitors on PI3K/AKT signalling depend on mutational status of the RAS/RAF signalling axis. Oncotarget.

[5] Fritsche-Guenther, R. *et al*. Strong negative feedback from Erk to Raf confers robustness to MAPK signalling. *Molecular systems biology* **7**, 489, doi:msb201127 (2011). 10.1038/msb.2011.27 PMC3130559 21613978

[6] Klinger B (2013). Network quantification of EGFR signaling unveils potential for targeted combination therapy. Molecular systems biology.

[7] Hanahan D, Weinberg RA (2011). Hallmarks of cancer: the next generation. Cell.

[8] Hutton JE (2016). Oncogenic KRAS and BRAF Drive Metabolic Reprogramming in Colorectal Cancer. Molecular & cellular proteomics: MCP.

[9] Gaglio D (2011). Oncogenic K-Ras decouples glucose and glutamine metabolism to support cancer cell growth. Molecular systems biology.

[10] Chiaradonna F (2006). Ras-dependent carbon metabolism and transformation in mouse fibroblasts. Oncogene.

[11] Liberti MV, Locasale JW (2016). The Warburg Effect: How Does it Benefit Cancer Cells?. Trends in biochemical sciences.

[12] Warburg O, Wind F, Negelein E (1927). The Metabolism of Tumors in the Body. The Journal of general physiology.

[13] Lenaerts K, Bouwman FG, Lamers WH, Renes J, Mariman EC (2007). Comparative proteomic analysis of cell lines and scrapings of the human intestinal epithelium. BMC genomics.

[14] Vander Heiden MG, Cantley LC, Thompson CB (2009). Understanding the Warburg effect: the metabolic requirements of cell proliferation. Science.

[15] Sambuy Y (2005). The Caco-2 cell line as a model of the intestinal barrier: influence of cell and culture-related factors on Caco-2 cell functional characteristics. Cell biology and toxicology.

[16] Bu XD, Li N, Tian XQ, Huang PL (2011). Caco-2 and LS174T cell lines provide different models for studying mucin expression in colon cancer. Tissue & cell.

[17] Jewell JL, Guan KL (2013). Nutrient signaling to mTOR and cell growth. Trends in biochemical sciences.

[18] Wan X, Harkavy B, Shen N, Grohar P, Helman LJ (2007). Rapamycin induces feedback activation of Akt signaling through an IGF-1R-dependent mechanism. Oncogene.

[19] Gulhati P (2009). Targeted inhibition of mammalian target of rapamycin signaling inhibits tumorigenesis of colorectal cancer. Clinical cancer research: an official journal of the American Association for Cancer Research.

[20] Eisenhardt AE (2016). Phospho-proteomic analyses of B-Raf protein complexes reveal new regulatory principles. Oncotarget.

[21] Bhagwat SV (2011). Preclinical characterization of OSI-027, a potent and selective inhibitor of mTORC1 and mTORC2: distinct from rapamycin. Molecular cancer therapeutics.

[22] Kang HB (2015). Metabolic Rewiring by Oncogenic BRAF V600E Links Ketogenesis Pathway to BRAF-MEK1 Signaling. Molecular cell.

[23] Pietzke M, Kempa S (2014). Pulsed stable isotope-resolved metabolomic studies of cancer cells. Methods in enzymology.

[24] Pietzke M, Zasada C, Mudrich S, Kempa S (2014). Decoding the dynamics of cellular metabolism and the action of 3-bromopyruvate and 2-deoxyglucose using pulsed stable isotope-resolved metabolomics. Cancer & metabolism.

[25] Halestrap AP, Price NT (1999). The proton-linked monocarboxylate transporter (MCT) family: structure, function and regulation. The Biochemical journal.

[26] Doherty JR (2014). Blocking lactate export by inhibiting the Myc target MCT1 Disables glycolysis and glutathione synthesis. Cancer research.

[27] Le Floch R (2011). CD147 subunit of lactate/H+symporters MCT1 and hypoxia-inducible MCT4 is critical for energetics and growth of glycolytic tumors. Proceedings of the National Academy of Sciences of the United States of America.

[28] Polanski R (2014). Activity of the monocarboxylate transporter 1 inhibitor AZD3965 in small cell lung cancer. Clinical cancer research: an official journal of the American Association for Cancer Research.

[29] Lardy HA, Wiebelhaus VD, Mann KM (1950). The mechanism by which glyceraldehyde inhibits glycolysis. The Journal of biological chemistry.

[30] Mendel, B. KREBSZELLE UND GLYCERINALDEHYD. *Klin*. *Wochschr*. (1929).

[31] Warburg O, Gawehn K, Geissler AW, Lorenz S (1963). [on Destruction of Cancer Cells with Roentgen Rays]. Zeitschrift fur Naturforschung. Teil B, Chemie, Biochemie, Biophysik, Biologie und verwandte Gebiete.

[32] She QB (2010). 4E-BP1 is a key effector of the oncogenic activation of the AKT and ERK signaling pathways that integrates their function in tumors. Cancer cell.

[33] Konstantakou EG (2015). 3-BrPA eliminates human bladder cancer cells with highly oncogenic signatures via engagement of specific death programs and perturbation of multiple signaling and metabolic determinants. Molecular cancer.

[34] Sun Y (2015). Mechanisms underlying 3-bromopyruvate-induced cell death in colon cancer. Journal of bioenergetics and biomembranes.

[35] Moschetta M, Reale A, Marasco C, Vacca A, Carratu MR (2014). Therapeutic targeting of the mTOR-signalling pathway in cancer: benefits and limitations. British journal of pharmacology.

[36] Romero-Garcia S, Lopez-Gonzalez JS, Baez-Viveros JL, Aguilar-Cazares D, Prado-Garcia H (2011). Tumor cell metabolism: an integral view. Cancer biology & therapy.

[37] Gwinn DM (2008). AMPK phosphorylation of raptor mediates a metabolic checkpoint. Molecular cell.

[38] Do K (2015). Biomarker-driven phase 2 study of MK-2206 and selumetinib (AZD6244, ARRY-142886) in patients with colorectal cancer. Investigational new drugs.

[39] Esteve-Puig R, Canals F, Colome N, Merlino G, Recio JA (2009). Uncoupling of the LKB1-AMPKalpha energy sensor pathway by growth factors and oncogenic BRAF. PloS one.

[40] Diedrich B (2017). Discrete cytosolic macromolecular BRAF complexes exhibit distinct activities and composition. The EMBO journal.

[41] Oshiro N (2004). Dissociation of raptor from mTOR is a mechanism of rapamycin-induced inhibition of mTOR function. Genes to cells: devoted to molecular & cellular mechanisms.

[42] Falcon BL (2011). Reduced VEGF production, angiogenesis, and vascular regrowth contribute to the antitumor properties of dual mTORC1/mTORC2 inhibitors. Cancer research.

[43] Blaser B (2012). Antitumor activities of ATP-competitive inhibitors of mTOR in colon cancer cells. BMC cancer.

[44] Faes S (2016). Acidic tumor microenvironment abrogates the efficacy of mTORC1 inhibitors. Molecular cancer.

[45] Rappsilber J, Ishihama Y, Mann M (2003). Stop and go extraction tips for matrix-assisted laser desorption/ionization, nanoelectrospray, and LC/MS sample pretreatment in proteomics. Analytical chemistry.

[46] Cox J, Mann M (2008). MaxQuant enables high peptide identification rates, individualized p.p.b.-range mass accuracies and proteome-wide protein quantification. Nature biotechnology.

[47] Cox J (2011). Andromeda: a peptide search engine integrated into the MaxQuant environment. Journal of proteome research.

[48] Bielow C, Mastrobuoni G, Kempa S (2016). Proteomics Quality Control: Quality Control Software for MaxQuant Results. Journal of proteome research.

[49] Tyanova S (2016). The Perseus computational platform for comprehensive analysis of (prote)omics data. Nature methods.

[50] Junker BH, Klukas C, Schreiber F (2006). VANTED: a system for advanced data analysis and visualization in the context of biological networks. BMC bioinformatics.

[51] Kuich PH, Hoffmann N, Kempa S (2014). Maui-VIA: A User-Friendly Software for Visual Identification, Alignment, Correction, and Quantification of Gas Chromatography-Mass SpectrometryData. Frontiers in bioengineering and biotechnology.

[52] Klipp E, Heinrich R, Holzhutter HG (2002). Prediction of temporal gene expression. Metabolic opimization by re-distribution of enzyme activities. European journal of biochemistry / FEBS.

[53] Lin SJ, Guarente L (2003). Nicotinamide adenine dinucleotide, a metabolic regulator of transcription, longevity and disease. Current opinion in cell biology.

[54] Yamada K, Hara N, Shibata T, Osago H, Tsuchiya M (2006). The simultaneous measurement of nicotinamide adenine dinucleotide and related compounds by liquid chromatography/electrospray ionization tandem mass spectrometry. Analytical biochemistry.

[55] Beis I, Newsholme EA (1975). The contents of adenine nucleotides, phosphagens and some glycolytic intermediates in resting muscles from vertebrates and invertebrates. The Biochemical journal.

[56] Raue A (2015). Data2Dynamics: a modeling environment tailored to parameter estimation in dynamical systems. Bioinformatics.

