# Supplementary material for: Alterations of mTOR signaling impact metabolic stress resistance in colorectal carcinomas with BRAF and KRAS mutations
Source: Sci Rep. 2018 Jun 15;8:9204. doi: 10.1038/s41598-018-27394-1 (PMC6003911; doi:10.1038/s41598-018-27394-1)
Supplement: Supplementary file 1 — Supplementary Table and Figures [file 41598_2018_27394_MOESM1_ESM.pdf]

Supplementary material for

Alterations to mTOR signaling impacts metabolic stress resistance in BRAF and KRAS mutated colorectal carcinomas

Raphaela Fritsche-Guenther[1], Christin Zasada[2], Guido Mastrobuoni[2], Nadine Royla[2], Roman Rainer[3], Florian Roßner[4], Matthias Pietzke[5], Edda Klipp[4], Christine Sers[4], Stefan Kempa[1, 2]*

[1]Max-Delbrueck-Center for Molecular Medicine in the Helmholtz Association (MDC), Berlin Institute of Health (BIH), Robert-Roessle-Str. 10, 13125 Berlin, Germany

[2]Max-Delbrueck-Center for Molecular Medicine in the Helmholtz Association (MDC), The Berlin Institute for Medical Systems Biology (BIMSB), Robert-Roessle-Str. 10, 13125 Berlin, Germany

[3]Humboldt University Berlin, Theoretical Biophysics, Invalidenstraße 42, 10115 Berlin, Germany

[4]Charité Universitätsmedizin, Institute of Pathology, Chariteplatz 1, 10117 Berlin, Germany

[5]Beatson Institute, Switchback Road, Bearsden, Glasgow, G61 1BD, United Kingdom

Supplementary Table

Supplementary Table 1: pSIRM approach was performed using CaCO2-control, CaCO2-BRAF^{V600E} and CaCO2-KRAS^{G12V} cells (in minimum n=3). Mean of quantities (pmol/L 000 000 cells), ^{13}C-glucose incorporation (LI [%]) and normalized labeled quantities (NLQ [pmol/L 000 000 cells]) of metabolites (product of quantities and incorporation). Cells were cultivated and labeled for 5 min with physiological amounts of glucose (1.0 g/L). Ala: Alanine, Cit: Citric acid, Fum: Fumaric acid, Lac: Lactic acid, Mal: Malic acid, Pyr: Pyruvic acid, Ser: Serine, Suc: Succinic acid.

	**Cell line**	**CaCO2-control**	**CaCO2-BRAF^{V600E}**	**CaCO2-KRAS^{G12V}**
**quantities**	**Ser**	18935	15076	71435
	**Pyr**	2805	969	2568
	**Lac**	41227	45932	32545
	**Ala**	4097	865	688
	**Cit**	2493	3110	4167
	**Suc**	1641	1766	3635
	**Fum**	763	901	1565
	**Mal**	1843	1189	4354
**LI**	**Ser**	0.3	0.7	0.4
	**Pyr**	20.9	24.3	26.4
	**Lac**	16.7	18.5	10.7
	**Ala**	3.3	6.3	5.4
	**Cit**	10.8	18.5	11.0
	**Suc**	9.1	9.2	9.3
	**Fum**	1.7	1.9	0.5
	**Mal**	13.6	14.2	13.2
**NLQ**	**Ser**	46	120	95
	**Pyr**	527	241	864
	**Lac**	6716	8538	3745
	**Ala**	98	60	35
	**Cit**	343	650	448
	**Suc**	160	190	377
	**Fum**	14	19	8
	**Mal**	262	180	629

Supplementary Figures

Supplement Figure 1: A) Cells were cultivated in the presence of Doxycycline and indicated glucose concentrations for 16 d. **B)** CaCO2-control, CaCO2-BRAF^{V600E} and CaCO2-KRAS^{G12V} cells were cultivated in medium containing 1.0 g/L glucose and measured with LC-MS (shot gun proteomics). Shown are log2 fold changes (fc) to CaCO2-control cells for proteins associated with epithelial to mesenchymal transition, migration and actin remodeling. Significant regulations ($p<0.05$ unpaired two-tailed *t* Test) comparing CaCO2-control and CaCO2-BRAF^{V600E} or CaCO2-control and CaCO2-KRAS^{G12V} cells were indicated with asterisks or crosses, respectively. **C)** CaCO2-control, CaCO2-BRAF^{V600E} and CaCO2-KRAS^{G12V} cells cultivated in medium containing physiological (1.0 g/L) glucose were stained with periodic acid-Schiff (PAS) and alcian blue (AB) to verify neutral and acidic Mucin expression in vacuoles, respectively. **D)** MUC5AC expression was analyzed using ELISA assay. Cells were cultivated in physiological (1.0 g/L) glucose. Data were quantified and shown as log2 fold changes (fc). B, C, E) Shown are standard deviation of n=3 replicates. $p<0.05$ was indicated with asterisk (unpaired two-tailed *t* Test).

Supplement Figure 2: A, B) Flow cytometry profiles of CaCO2-control, CaCO2-BRAF^{V600E}, CaCO2-KRAS^{G12V}, HT29 and SW480 cells cultivated with indicated glucose amounts stained for cleaved Caspase 3. Shown are percent (%) of apoptotic cells. Shown are standard of n=2 replicates. $p<0.05$ was indicated with asterisk (unpaired two-tailed *t* Test)). **C, D)** CaCO2-control, CaCO2-BRAF^{V600E}, CaCO2-KRAS^{G12V}, HT29 and SW480 cells grown in physiological (1.0 g/L) glucose concentrations were treated with 10 µM Rapamycin (+), 10 µM OSI027 (+) or DMSO (-) for 24 h and analyzed with antibody against phosphorylated 4eBP1 (Thr70). Vinculin served as loading control. Samples for each cell line were loaded on separate gels. **E, H)** Phosphorylation of S6-kinase after treatment with 10 µM Rapamycin (+), 10 µM OSI027 (+) or DMSO (-) and AKT after treatment with 1 µM (+) MK2206 or DMSO (-) for 24 h was analyzed with ELISA bead-based phosphoproteomics technology (BioPlex). Shown are log2 fold changes (fc) to DMSO control (per cell line). **F, G**) CaCO2-control, CaCO2-BRAF^{V600E}, CaCO2-KRAS^{G12V}, HT29 and SW480 cells grown in physiological glucose concentrations

were treated with 1 µM MK2206 (+) or DMSO (-) for 24 h. Shown are viable cells compared to DMSO. Shown are standard deviation of n=3 replicates. $p<0.05$ was indicated with asterisk (unpaired two-tailed *t* Test). **I, J)** CaCO2-control, CaCO2-BRAF^{V600E}, CaCO2-KRAS^{G12V}, HT29 and SW480 cells grown in physiological (1.0 g/L) glucose concentrations were treated with 1 µM MK2206 (+) or DMSO (-) for 24 h and analyzed with antibody against phosphorylated 4eBP1 (Thr70). Vinculin served as loading control. Samples for each cell line were loaded on separate gels. **G)** BRAF was immunoprecipitated and the immuncomplexes were blotted using antibodies against BRAF and RAPTOR (different plots). IP and lysates were loaded on different gels. Vinculin served as loading control.

Supplement Figure 3: A) Hierarchical clustering of relative protein quantities (z-score) were shown for CaCO2-control, CaCO2-BRAF^{V600E} and CaCO2-KRAS^{G12V} cells cultivated in physiological (1.0 g/L) glucose. Proteins associated to central carbon metabolism were indicated per cluster. Enrichment analysis was done using gene ontology biological process terms. **B-D)** CaCO2-control, CaCO2-BRAF^{V600E} and CaCO2-KRAS^{G12V} cells were exposed to 1.0 g/L (B, C) or 2.5 g/L (D) of ^{13}C-glucose for 5 min, harvested and measured with GC-MS. The ratio (log 2 fold changes, fc) of B) CaCO2-BRAF^{V600E} to CaCO2-control, C) CaCO2-KRAS^{G12V} to CaCO2-control or D) CaCO2-KRAS^{G12V} to CaCO2-BRAF^{V600E} for labeled (metabolites) quantities are shown. **E**) CaCO2-control, CaCO2-BRAF^{V600E} and CaCO2-KRAS^{G12V} cells were exposed to ^{13}C-glucose (physiological and intermediate amounts) for 5 min, harvested and measured with GC-MS. Extracellular lactic acid quantities were depicted. Shown are standard deviation of n=3 replicates. $p<0.05$ was indicated with asterisk (unpaired two-tailed *t* Test).

Supplement Figure 4: A) CaCO2-control, CaCO2-BRAF^{V600E} and CaCO2-KRAS^{G12V} cells were cultivated with indicated glucose concentrations and analyzed with quantitative real time PCR for *MCT1* and *MCT4* expression. *PGK1* served as loading control. Relative mRNA expression to CaCO2-control cultured with 1.0 g/L glucose was shown. **B, C)** CaCO2-control, CaCO2-BRAF^{V600E}, CaCO2-KRAS^{G12V}, HT29 and SW480 cells cultivated in medium containing 1.0 g/L or 2.5 g/L glucose

concentrations and treated with 0.1 µM SR13800 for 24 h were analyzed using quantitative real time PCR for *MCT1* and *MCT4* expression. *PGK1* served as loading control. Relative mRNA expression to DMSO was shown. glc: glucose. A-C) Shown are standard deviation of (in minimum) n=3 replicates. $p<0.05$ was indicated with asterisk (unpaired two-tailed *t* Test).

Supplement Figure 5: A) CaCO2-control, CaCO2-BRAF^{V600E} and CaCO2-KRAS^{G12V} cells were subcutaneously injected into the right flank of mice in the presence of Doxycycline (Dox) every 2 d by intraperitoneal injection. Mean of tumor volume is shown for n=3 mice per group up to 14 d. **B)** CaCO2-KRAS^{G12V} were subcutaneously injected into the right flank of mice receiving 200 µL PBS or BrPy (8 mg/kg) in the presence of Doxycycline treatment every 2 days (starting from day 8) by intraperitoneal injection. Hematoxylin eosin staining (H&E) staining was performed from paraffin embedded sections.

Supplementary Information for blots:

Full length blot to Figure 3A

Full length blot to Figure 3B

Full length blot to Figure 6D

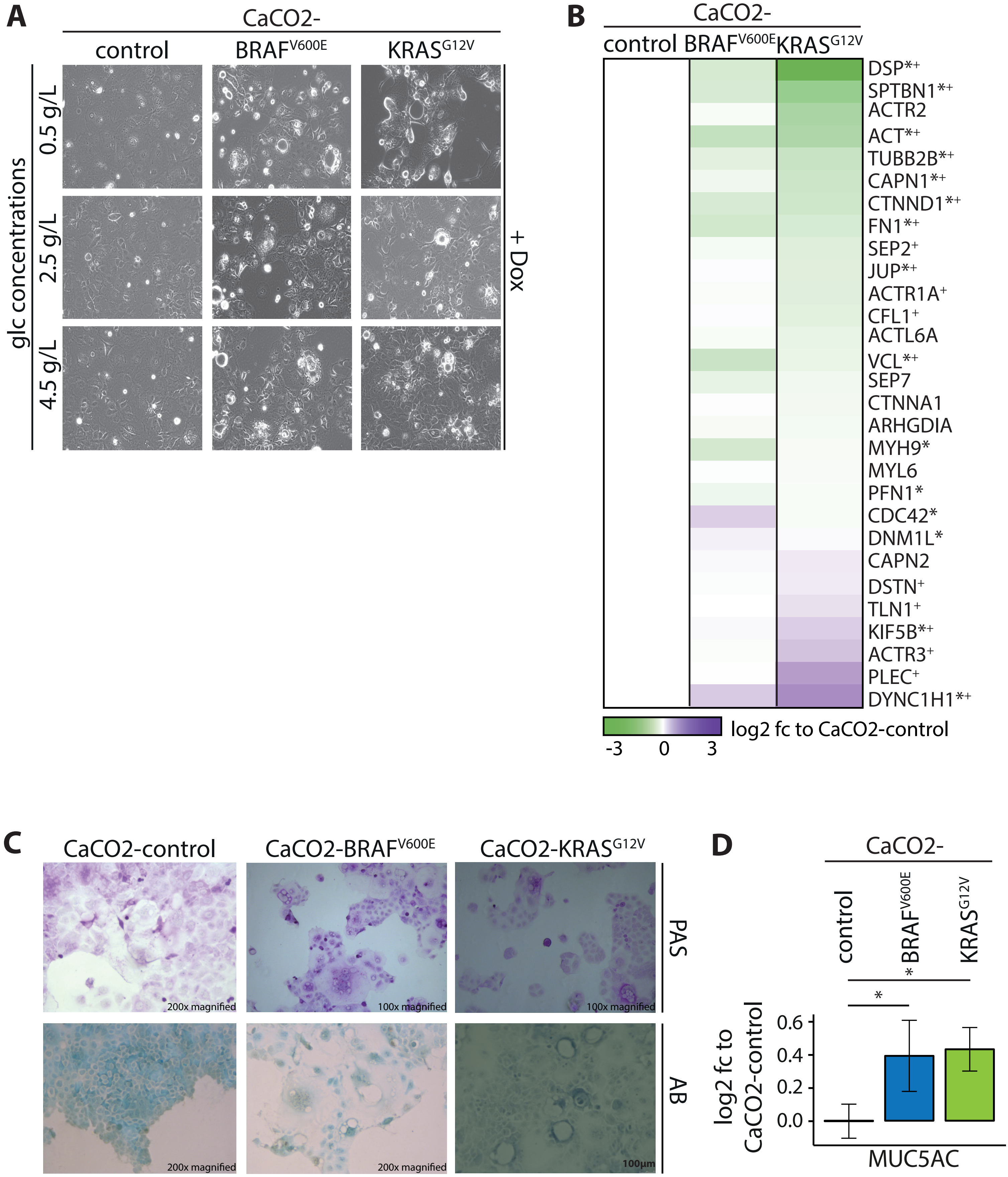

Supplement Figure 1

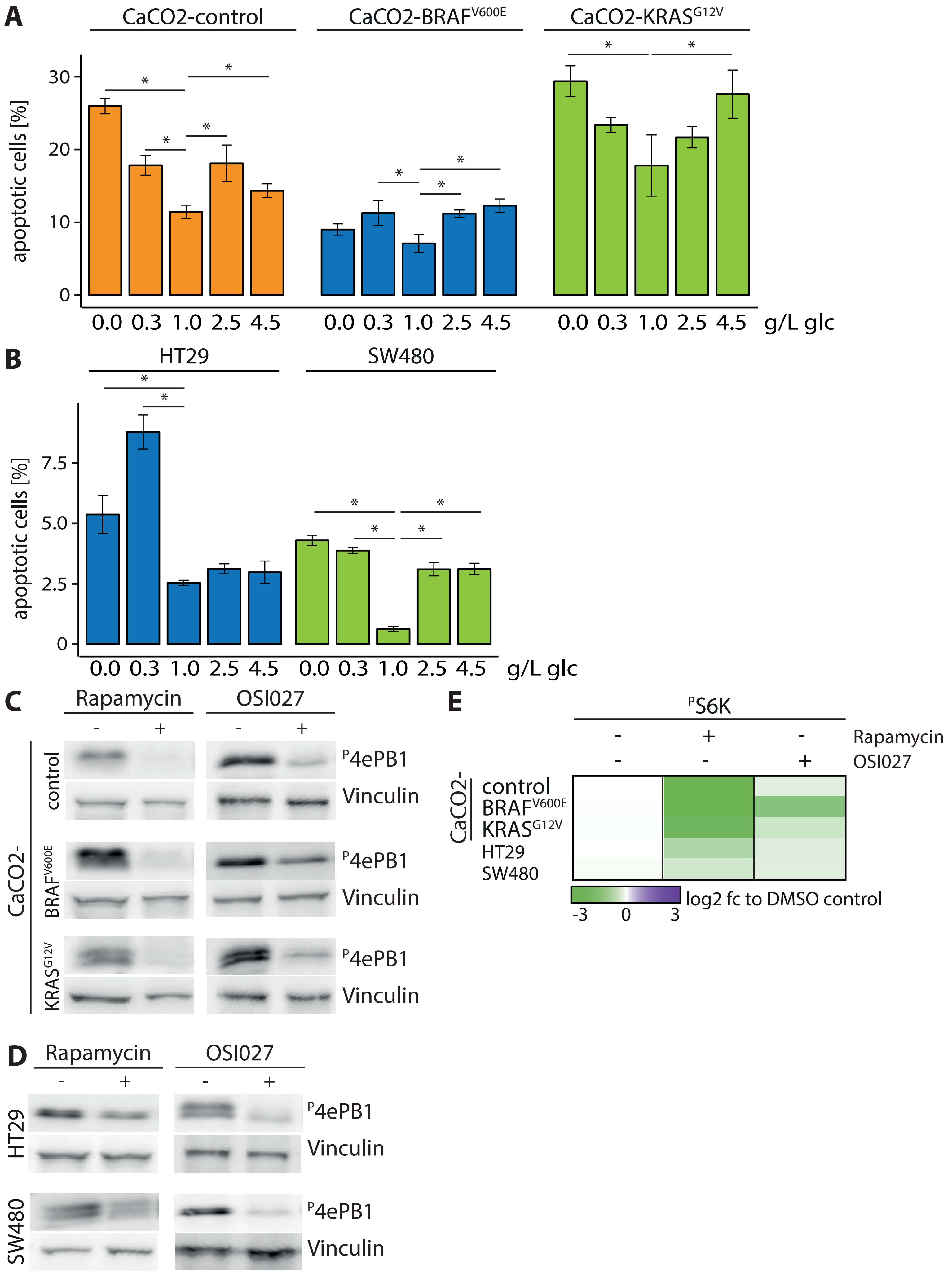

Supplement Figure 2

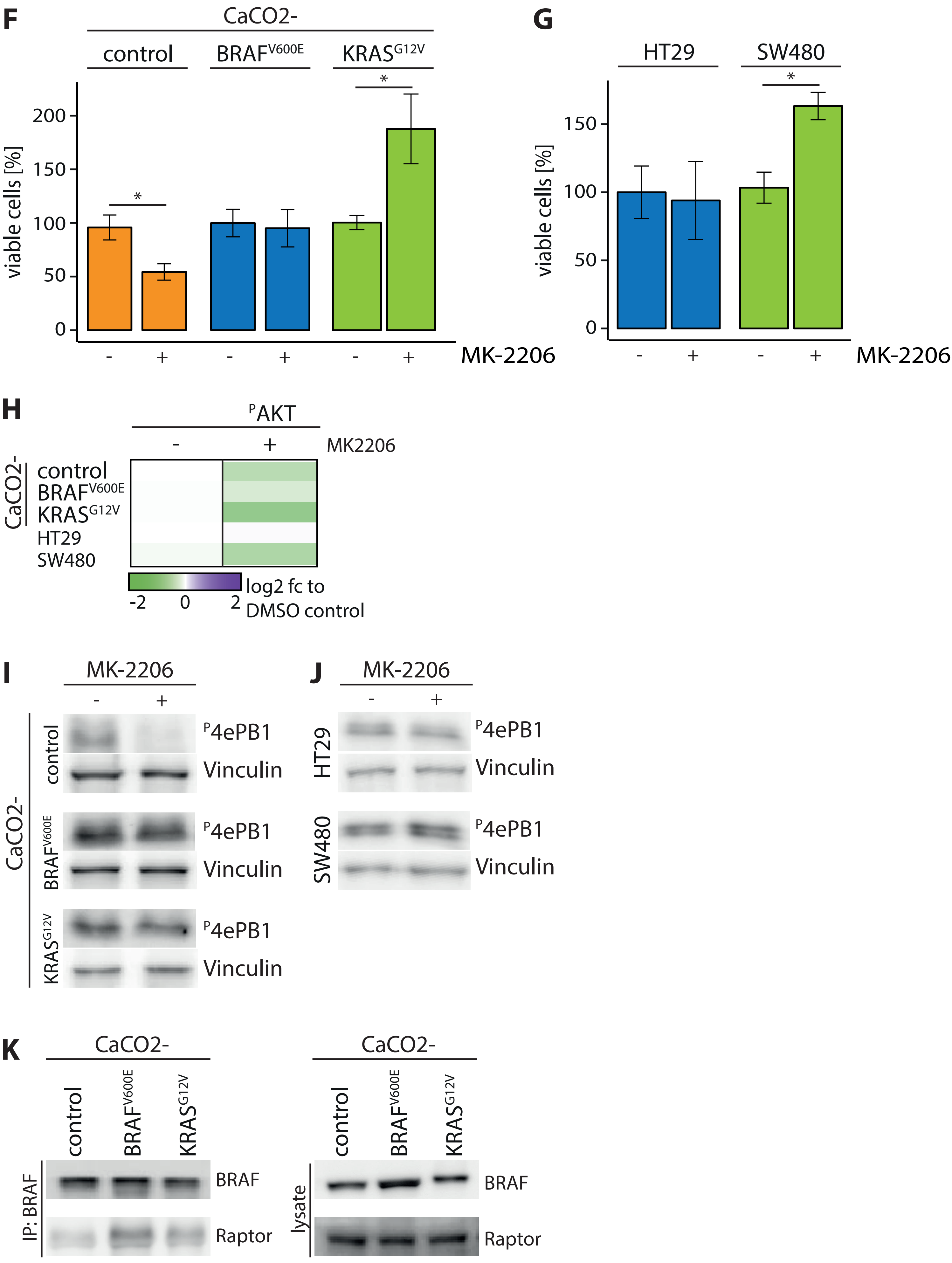

Supplement Figure 2

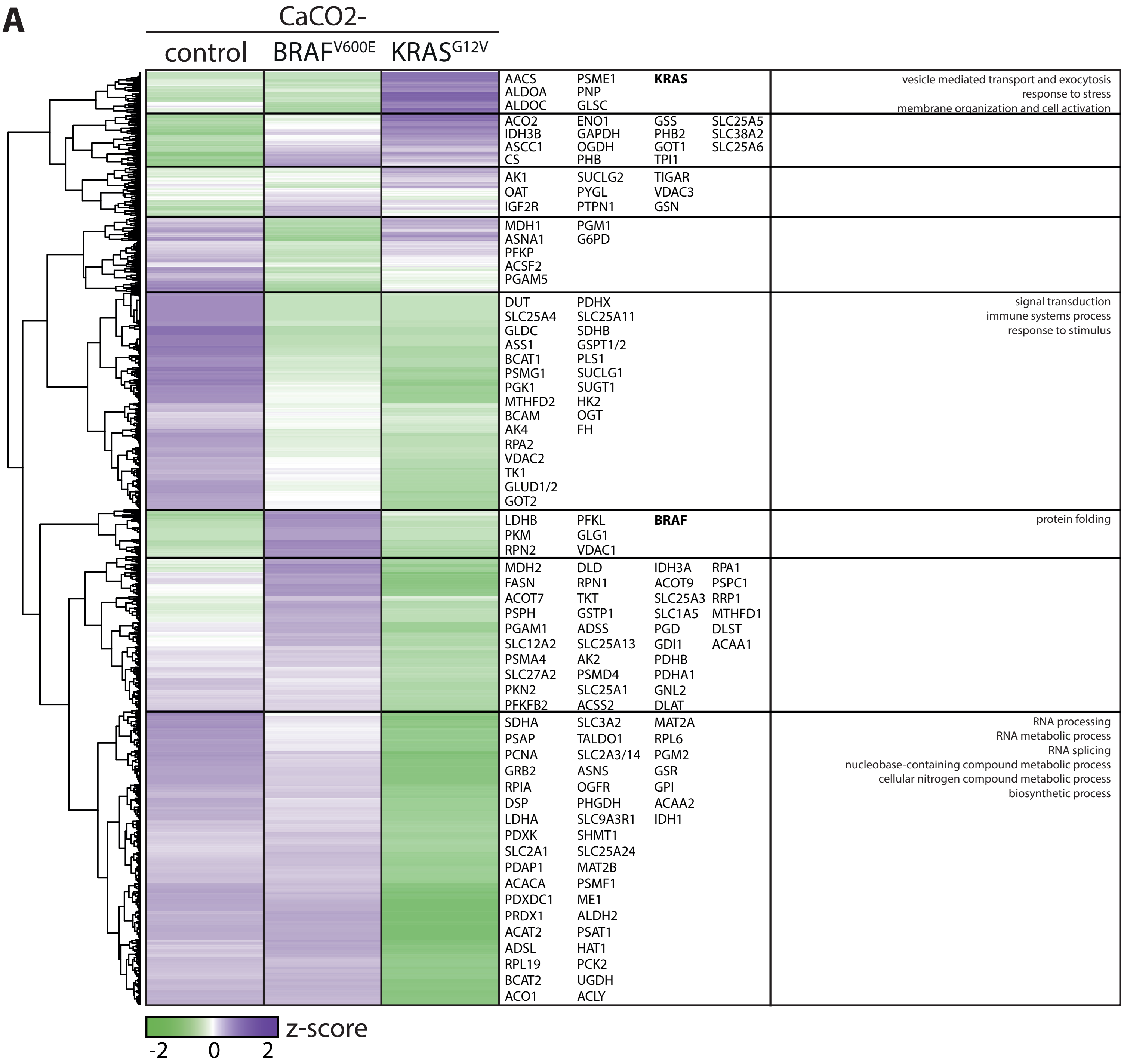

Supplement Figure 3

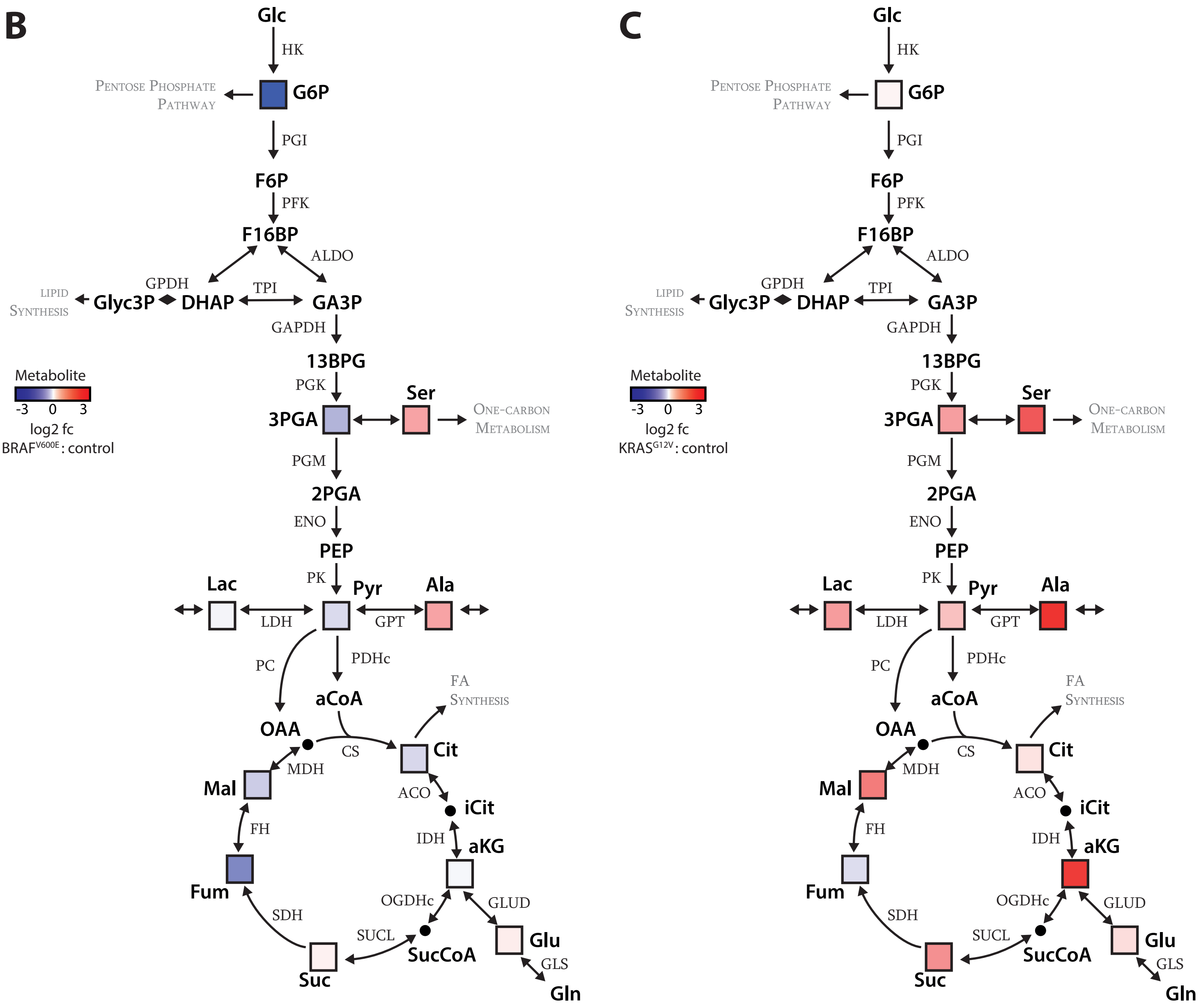

Supplement Figure 3

D

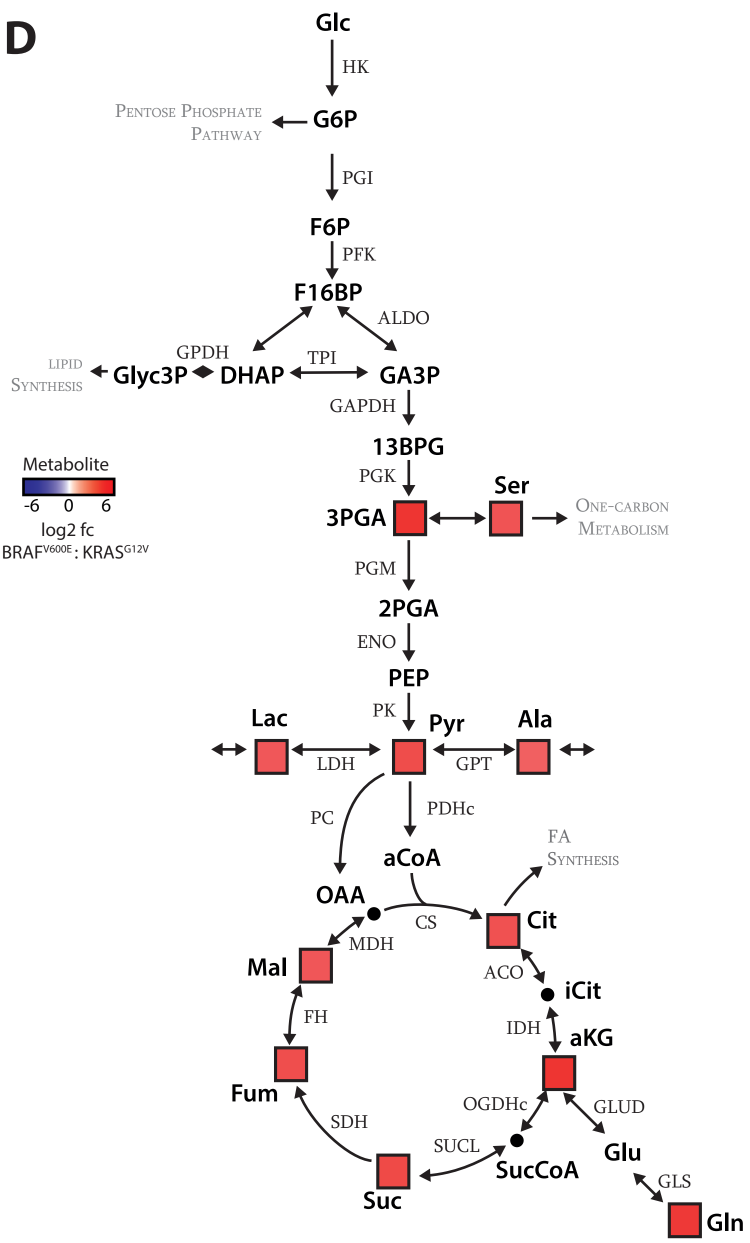

E

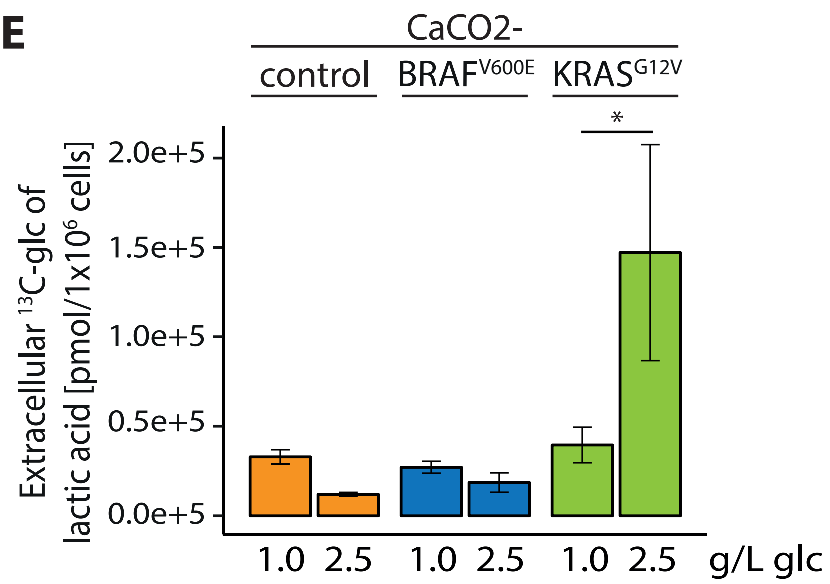

Supplement Figure 3

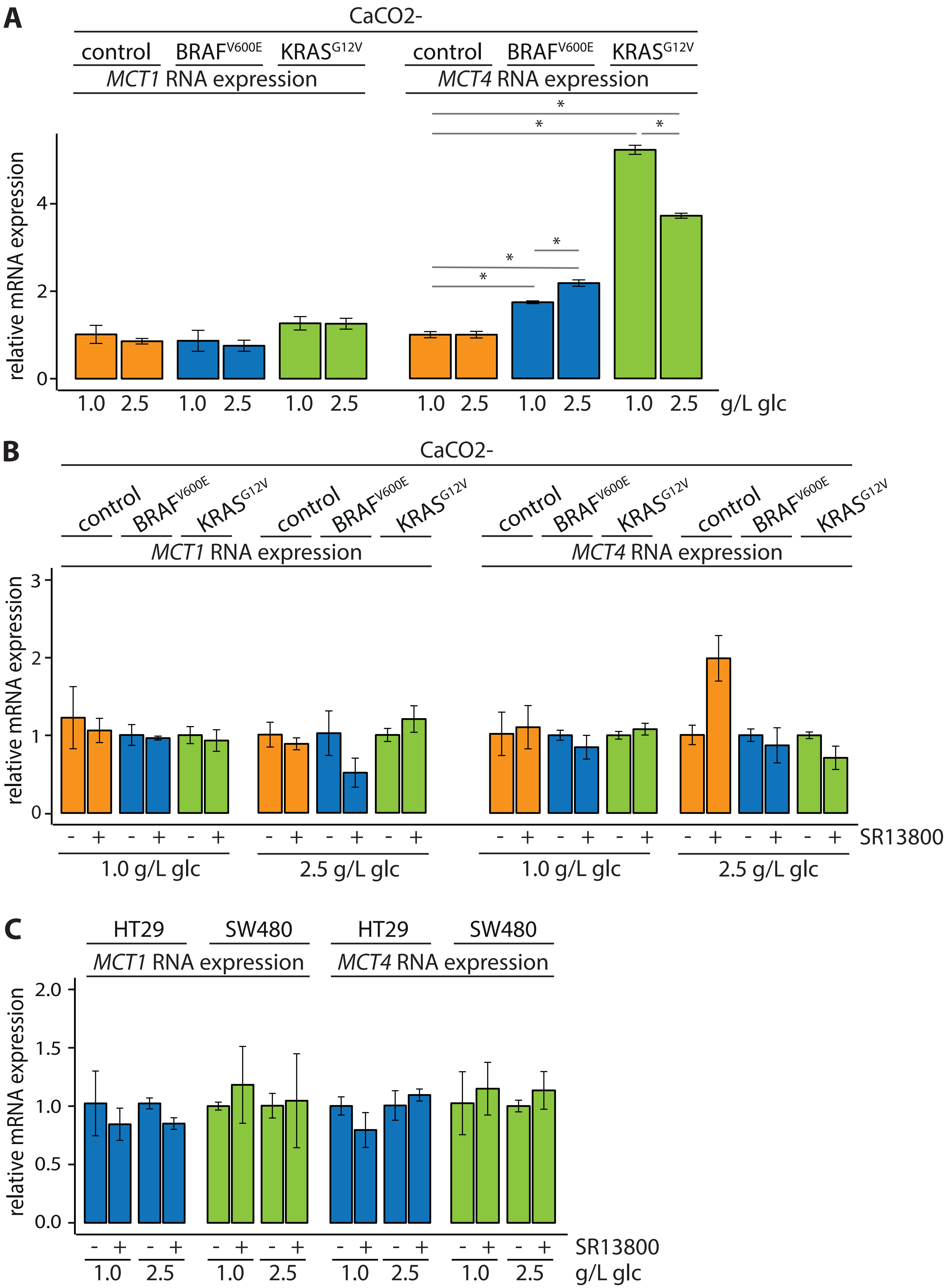

Supplementary Figure 4

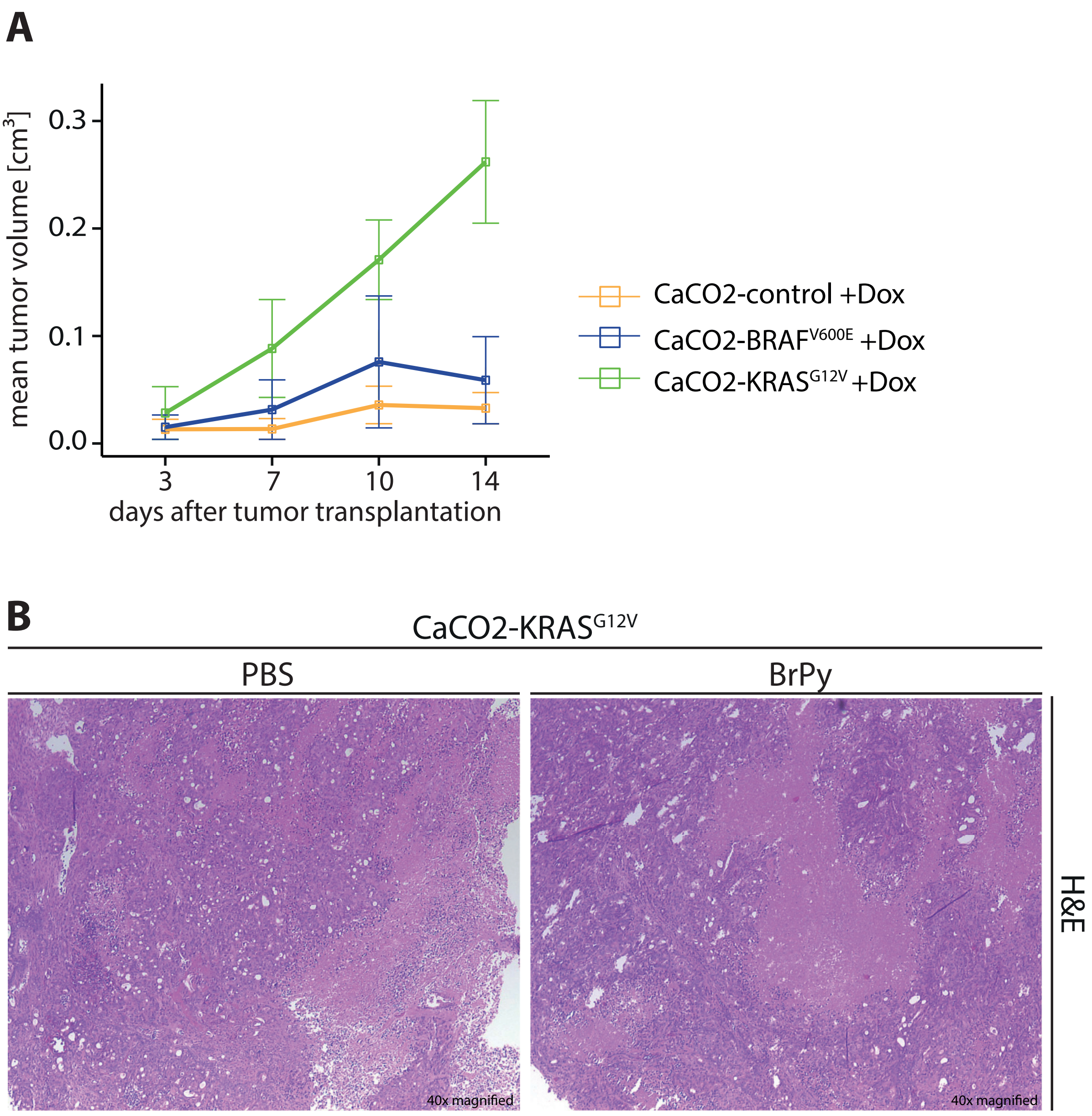

Supplementary Figure 5

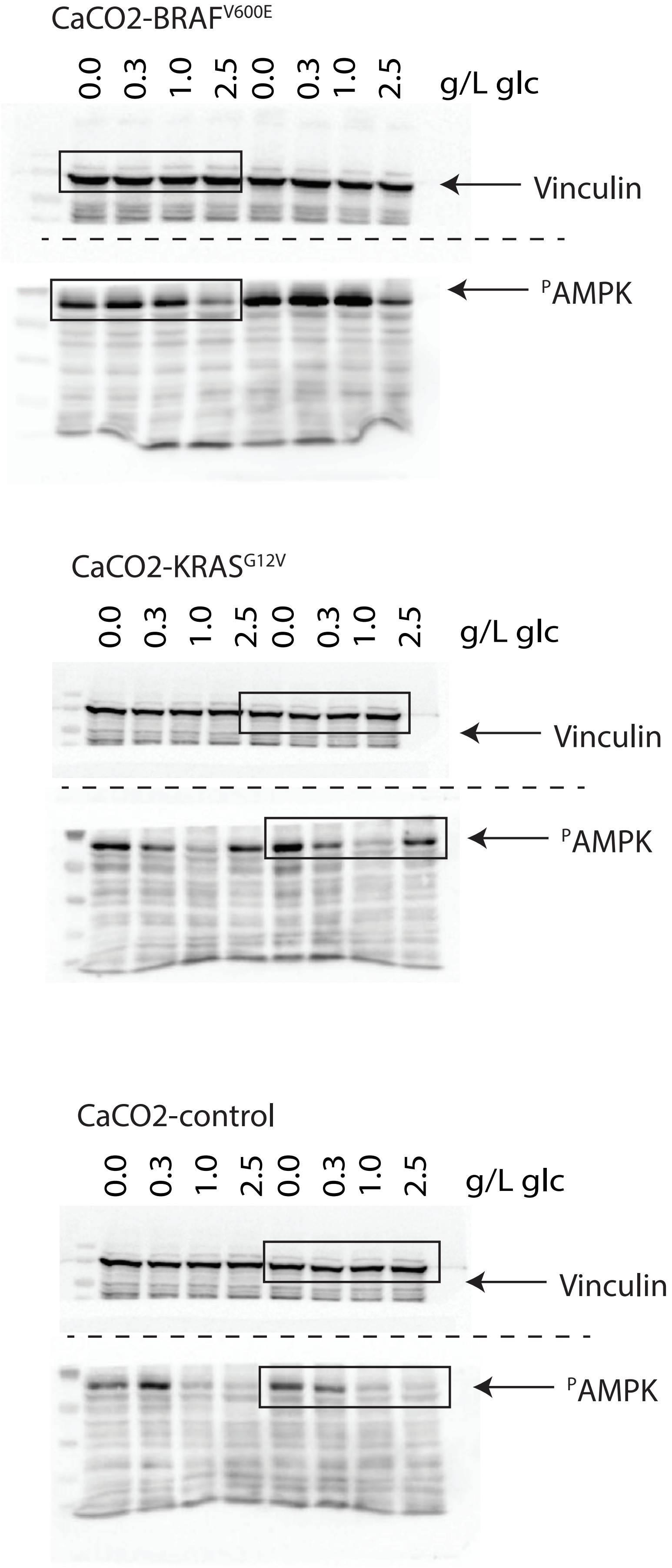

Full length blots to Figure 3A

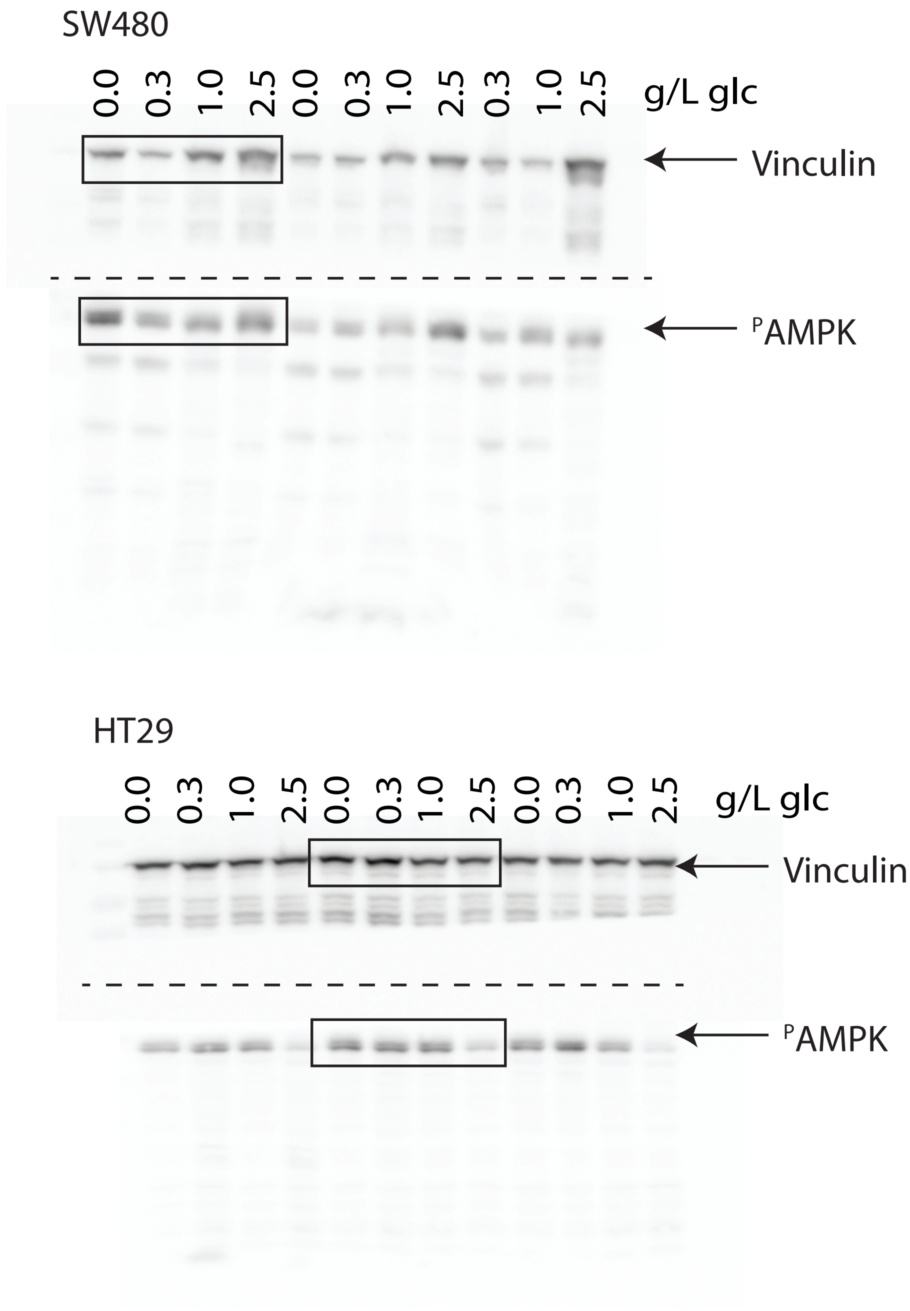

Full length blots to Figure 3B

CaCO2-BRAF^{V600E}

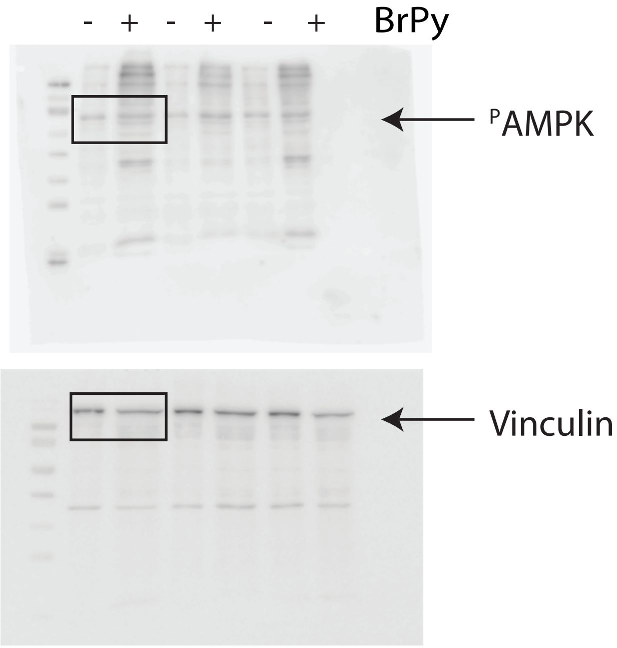

CaCO2-KRAS^{G12V}

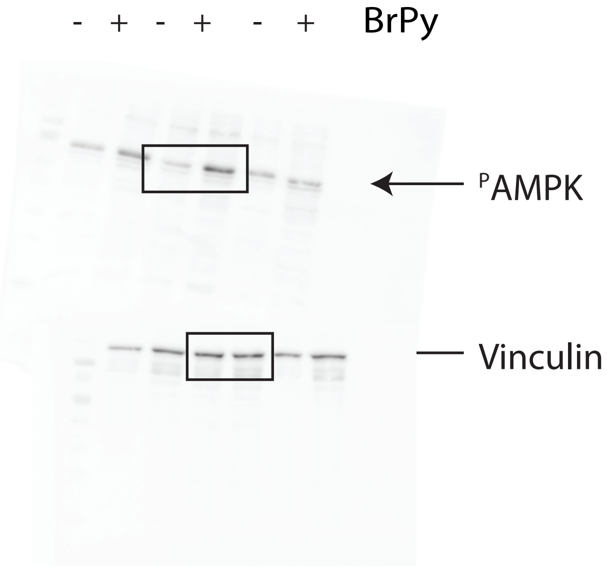

Full length blots to Figure 6D
